# Neuro-Particle Swarm Optimization Based In-Situ Prediction Model for Heavy Metals Concentration in Groundwater and Surface Water

**DOI:** 10.3390/toxics10020095

**Published:** 2022-02-18

**Authors:** Kevin Lawrence M. De Jesus, Delia B. Senoro, Jennifer C. Dela Cruz, Eduardo B. Chan

**Affiliations:** 1 School of Graduate Studies, Mapua University, Manila 1002, Philippines; klmdejesus@mymail.mapua.edu.ph (K.L.M.D.J.); jcdelacruz@mapua.edu.ph (J.C.D.C.); 2 School of Chemical, Biological, Materials Engineering and Sciences, Mapua University, Manila 1002, Philippines; 3 Resiliency and Sustainable Development Center, Yuchengco Innovation Center, Mapua University, Manila 1002, Philippines; 4 School of Civil, Environmental and Geological Engineering, Mapua University, Manila 1002, Philippines; 5 School of Electrical, Electronics and Computer Engineering, Mapua University, Manila 1002, Philippines; 6 Dyson College of Arts and Science, Pace University, New York, NY 10038, USA; echan@pace.edu

**Keywords:** groundwater, surface water, heavy metals, neural network, particle swarm optimization

## Abstract

Limited monitoring activities to assess data on heavy metal (HM) concentration contribute to worldwide concern for the environmental quality and the degree of toxicants in areas where there are elevated metals concentrations. Hence, this study used in-situ physicochemical parameters to the limited data on HM concentration in SW and GW. The site of the study was Marinduque Island Province in the Philippines, which experienced two mining disasters. Prediction model results showed that the SW models during the dry and wet seasons recorded a mean squared error (MSE) ranging from 6 × 10^−7^ to 0.070276. The GW models recorded a range from 5 × 10^−8^ to 0.045373, all of which were approaching the ideal MSE value of 0. Kling–Gupta efficiency values of developed models were all greater than 0.95. The developed neural network-particle swarm optimization (NN-PSO) models for SW and GW were compared to linear and support vector machine (SVM) models and previously published deterministic and artificial intelligence (AI) models. The findings indicated that the developed NN-PSO models are superior to the developed linear and SVM models, up to 1.60 and 1.40 times greater than the best model observed created by linear and SVM models for SW and GW, respectively. The developed models were also on par with previously published deterministic and AI-based models considering their prediction capability. Sensitivity analysis using Olden’s connection weights approach showed that pH influenced the concentration of HM significantly. Established on the research findings, it can be stated that the NN-PSO is an effective and practical approach in the prediction of HM concentration in water resources that contributes a solution to the limited HM concentration monitored data.

## 1. Introduction

The Philippines is an archipelagic country consisting of more than 7600 islands in the Southeast Asian region. The Philippines is among the countries in the world with vast mineral deposits, and it was assessed that thirty percent of the land area of the country has the resources to commence mining activities [1]. Although the mining industry provides immense livelihood opportunities, mining activities in the Philippines have a mixed footprint of economic progress and impact on humans and the environment. Consequently, regular environmental quality monitoring, especially heavy metal (HM) concentration, is lacking due to access to equipment, laboratory facilities, and water resource locations to regularly monitor the degree of HM concentration. These resources could lead to different risks, as much evidence is available that proper education and information regarding HM concentrations in water resources are essential. Lack of information on the quality of water could result in several detrimental effects not only on the environment but also on the health of the people in the community [2]. The list of abbreviations and symbols in this study is presented in Table 1.

Numerous areas affected by mine tailings have been identified in the Philippines. These mine tailings, normally called acid mine drainage (AMD), are primarily in remote areas and regions where access is a challenge. Due to vast deposits of minerals in the Philippines, mining activities commenced in several provinces of the archipelago. Some of these activities resulted in detrimental and extreme mining disasters that released toxic substances into the environment, affecting the ecosystem and the community adjacent to the mining site [3]. The summary of the mining disasters in the Philippines is presented in Table A1 in Appendix A.

Due to the inadequacies in environmental quality assessment, individuals lack awareness and understanding of the hazards presented by heavy metals to people, especially in low-income countries such as the Philippines [4]. Nearly three decades of mining tailings discharge on Marinduque Island have resulted in higher levels of HM in the environment and residents. The typical exposure pathways of Marinduque villagers residing near the Boac and Mogpog river, where mine tailings were hosted in 1993 and 1996, were bathing, laundering, and passing through the river without protective gear or equipment [5].

Heavy metal contamination in water resources is associated with high toxicity, permanence, and resistance to degradation, disrupting the regular function of water resource systems and constituting a hazard to people by accessing various routes and developing adverse significant health problems [6]. Exposure to these heavy metals can lead to many diseases, including kidney problems such as proteinuria from cadmium exposure [7]; lung carcinoma from chromium exposure [8]; severe damage to the nervous and reproductive system, kidneys, and liver from lead exposure [9]; manganism, which is described as a Parkinson’s-like syndrome that manifests weakness, myalgia, anorexia nervosa, apathy, slow speech, emotionless facial expression, monotonous tone of voice, and clumsy limb movement from manganese exposure [10]; contact dermatitis and eczema and respiratory problems from oral exposure to nickel [11]; damage to the liver, brain, and kidney from long-term copper exposure [12]; tissue damage, organ failure, and elevated cancer risks from exposure to high concentrations of iron [13]; risk of developing anemia and damage to the pancreas from chronic toxicity of zinc [14].

Testing of groundwater (GW) and surface water (SW) samples is a tedious activity due to the different phases involved such as collection, transport, treatment, storage, and analysis of samples. Another issue that might be encountered is the high cost involved in the elemental analysis of these water samples. An additional challenge encountered by the researchers in Marinduque Province is the difficulty in the procurement period for reagents, chemical, and calibration standard solutions that could take 3–4 months to receive, as there are imported from other countries. Another factor affecting the deficiency in monitoring water resources affected by AMD is insufficient skilled personnel to perform the periodic collection and testing of water samples. This condition has been experienced by various local government units (LGUs). Moreover, although inductively coupled plasma (ICP) and atomic absorption spectroscopy (AAS) instruments are being used effectively for heavy metal detection in aquatic environments, several disadvantages, such as expensive instrumentation, limited sample throughput, slow turnaround time, difficulty in performing required measurements for in-situ samples, and consumption of chemicals, have restricted their frequent use in affected mine tailing sites [15,16]. This situation prompted researchers to look for equally viable approaches to serve the need for convenient and regular water quality assessment and monitoring.

The use of innovative tools and methods is essential to address the current issues on environmental quality monitoring, especially in areas where there are AMDs. The onset of the industry 4.0 scenario gives birth to artificial intelligence (AI) tools and techniques that include the Internet of Things (IoT), smart sensors, advanced robotics, big data analytics, 3D printing, augmented reality, location detection, cloud computing, and machine learning (ML). Due to the input and output correlation capability of ML, it can be used as a possible approach for addressing the deficiency of instruments used for elemental analysis and tools to determine the concentration of the metals in surface and groundwater [17].

Machine learning is a data analytics technology that enables computers to learn from their experiences. It makes use of computational tools to derive knowledge completely from information, rather than depending on a predefined equation as a model. ML techniques discover natural patterns in data, providing insight and assisting in making more accurate judgments and predictions [18]. The transition to industry 4.0, including the rise in big data, enables the use of ML to become an essential technique in problem-solving of different areas and industries, including environmental quality monitoring [19]. The ML methods are classified into two categories: supervised and unsupervised learning. Whereas supervised learning is used to train a model on identified input and output datasets to forecast future outputs, unsupervised learning is used to discover unknown patterns or inherent structures in the input dataset. Supervised and unsupervised machine learning techniques are used concurrently. Classification and regression are part of supervised learning, whereas clustering is part of unsupervised learning. Support vector machines (SVM), discriminant analysis, Naive Bayes, and nearest neighbor are all examples of classification techniques, whereas multiple linear regression (MLR), generalized linear models, support vector regression (SVR), Gaussian process regression, ensemble methods, decision trees, and neural networks are all examples of regression techniques. The clustering approaches under unsupervised learning include hierarchical clustering, Markov models, and neural networks. Neural networks are classified as both supervised and unsupervised machine learning under the regression and clustering category.

The utilization of artificial neural networks (ANN) can capture the dominant characteristics of complex and non-linear systems. Neural networks apply a modeling method that is based on empirical data rather than on simplified ideal assumptions. ANN is broadly applied in various areas of environmental engineering such as air quality monitoring [20], soil quality monitoring [21], and water quality prediction [22]. The use of ANN was found to be superior in performance as it was implemented in various fields including medicine [23,24,25], geotechnical engineering [26], petroleum engineering [27], construction technology [28], building energy [29], and waste management [30], as compared to other models, such as linear and SVM models. Moreover, it was also found out that integrating an optimization technique to ANN further improved its performance as compared to linear and SVM models as observed in the studies of Sun et al. in 2019 [31] and Zhang et al. in 2020 [32].

Neural networks are a heuristic modeling technique that is established on the behavior of genetic neural configurations. ANN could eliminate the tedious and invasive process of digestion of water samples to obtain heavy metal concentrations [33,34]. By combining the input and output data, it is possible to construct a network of interconnected neurons capable of predicting variables given a set of inputs. The simplest and most broadly employed type of neural network is the feed-forward multilayered backpropagation algorithm (BP-NN). However, traditional algorithms such as BP-NN have limitations such as being unable to traverse error function peaks.

To address the BP-NN difficulties, evolutionary algorithms optimization methods such as particle swarm optimization (PSO) are used. PSO has a global search function that could be utilized to optimize the performance of the BP-NN [35]. The PSO method is a well-established optimization technique that draws inspiration from the group interactions and motion patterns of insects, birds, and fish. It is a search method based on optimization and stochastic population search that seeks to solve problems in a continuous solution space [36].

Recent studies involved the use of different ML tools in predicting heavy metals in AMD-affected surface and groundwater sites. Table 2 presents the machine learning models from published papers predicting heavy metal concentrations in surface and groundwater.

These ML models for heavy metal detection were developed using water parameters that can only be obtained through laboratory testing such as nitrate, sulphate, biological oxygen demand (BOD), chemical oxygen demand (COD), total nitrogen (TN), total phosphorus (TP), and phosphate, to name a few. The in-situ implementation has never been carried out due to due to the challenges described above. Therefore, the purpose of this study was to develop a heavy metal prediction model that utilized in-situ measurements to create tools, such as prediction models, to address the challenges in conducting regular water quality assessment and monitoring.

## 2. Materials and Methods

The study implemented a hybrid neuro–particle swarm optimization (hN-PSO) approach in predicting heavy metal concentrations in SW and GW. This hN-PSO method utilized simple physicochemical water properties such as temperature, water pH, electrical conductivity (EC), and total dissolved solids (TDS) as input datasets. The number of water samples with elemental analysis was also included. These SW and GW samples were utilized to generate spatial concentration maps of physicochemical parameters and heavy metal concentrations using the machine learning geostatistical interpolation (MLGI) approach through the use of MATLAB and GIS ArcMap. The following sections detail the focus and analysis implemented in this study.

### 2.1. Location of the Study and Data Gathering

**Figure 1:**
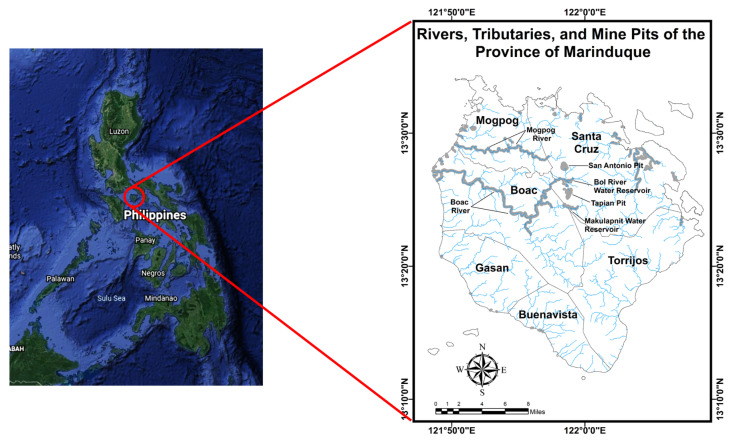
Major rivers and its tributaries in the province of Marinduque.

One of the largest mining activities in the country was in the province of Marinduque, an island in the southern portion of Luzon approximately 160 km south of Metro Manila. The mining operations in the province started in 1969 and the generated mine tailings were discharged to the major water bodies on the island [59]. An earth dam was constructed at Maguilaguila to prevent silt from a waste pit from being discharged to the Mogpog River. A dam break happened in 1993 and impacted the Mogpog River, causing detrimental effects such as flooding, significant adverse effects on the infrastructure, agriculture, and public health in the surrounding community. In 1996, the Tapian Pit collapsed, dumping approximately 180,000 to 260,000 m^3^ of mine tailings into the 27-km-long Boac River. This was considered one of the world’s worst mining disasters [60]. The rivers and tributaries in the province of Marinduque were shown in Figure 1.

The GW samples were collected from various wells from six municipalities of Marinduque island province. Surface water samples were also taken from the island province’s different rivers and tributaries. One (1) liter polyethylene bottle was used for samples collection. The GW and SW data were gathered in accordance with EPA regulations SESDPROC-301-R3 [61] and SESDPROC-201-R3 [62]. The SW bodies in the province of Marinduque are classified as Type C, which is defined as fishery water for the reproduction as well as the growth of aquatic species, for boating, fishing, and water for agriculture, according to the Department of Environment and Natural Resources (DENR) Administrative Order (DAO) 2016–08 [63]. The island province has a climatic classification of Type III, with a Dry Season (DS) running from November to April and a Wet Season (WS) spanning the remainder of the year [64]. Both GW and SW samples were collected covering the DS and WS.

### 2.2. Analysis of Physicochemical Parameters and HM Concentrations

In–situ measurements of the physicochemical parameters from 62 SW and 34 GW sampling sites were collected during the DS, whereas 59 SW and 49 GW were collected during the WS. These water properties were temperature (°C), pH, EC (µS/cm), and TDS (mg/L) using a Hanna HI 9811-5 handheld multi-parameter sampler. Total metals were also quantified for each surface water and groundwater sample. The EPA Method 3005A and 200.7 were used for the acid digestion and elemental analysis, respectively, to determine total recoverable or dissolved metals [65,66] and HM metal concentrations for SW and GW at limited sampling locations. The elemental analysis was carried out by ICP-OES Optima 8000.

### 2.3. Neuro-Particle Swarm Optimization Modelling (NN-PSO)

The prediction models for the HM concentration in SW and GW were developed using a particle swarm optimization informed backpropagation neural network. For this purpose, the MATLAB R2021a Neural Network Toolbox was utilized throughout the study.

#### 2.3.1. Machine Learning Geostatistical Interpolation (MLGI) Mapping

The collected surface water and groundwater samples during the dry season (DS) and wet season (WS) were mapped using an MLGI method [67]. This included physicochemical parameters and heavy metal concentrations of SW and GW. The hybrid technique integrated with the empirical Bayesian kriging (EBK) method generated the spatial concentration maps of the target study area. The generated maps were used as part of the datasets utilized in the development of heavy metal prediction models.

#### 2.3.2. Data Pre-Processing

The ANN is capable of processing purely numerical data input. As a result, all data must be transformed into numerical data input format. Data normalization was accomplished by scaling the network’s input and output data nodes between −1 and +1. To prevent the inverse effect of input variables with differing scales, the data from the input variables and heavy metal concentrations were normalized to the same scale. Data normalization was used to ensure rapid convergence and to acquire the lowest mean square error (MSE) values possible [45]. The normalized values of each input and output were achieved using Equation (1).

(1) $$y=\frac{\left(y_{max}-y_{min}\right)\left(x-x_{min}\right)}{\left(x_{max}-x_{min}\right)}+y_{min}$$

where *y* is the normalized value, *y_max_* = +1, *y_min_* = −1, *x* is the real value, and *x_max_* and *x_min_* are the upper and lower limit quantities of the parameter being normalized.

#### 2.3.3. Backpropagation Neural Network

ANN is a basic and appropriate approach for modeling non-linear connections. To describe the effects of each aspect, it is based on a mathematical model that incorporates managing components called neurons and the relationships between them. This approach’s extensive flexibility to generate outcomes from complex or partial data makes it ideal for forecasting unique scenarios [68]. ANN is a data management strategy that mimics how a natural neural system like the human brain integrates data. It exports experimental data to the network configuration. The network understands the total system by calculating mathematical facts. The model’s key feature is its unique data management structure arrangement. An ANN is designed to categorize data or identify arrays using knowledge-based techniques. The synaptic interactions between neurons include learning knowledge [69]. The learning methodology is used to change and develop the neural network. The model is upgraded to provide a better result from a given input. An ANN is composed of three layers, each of which is derived from a biological neuron. Input neurons (IN) of the biological neuron transmit data to the hidden layer (HL), which in turn delivers data to the output neurons (ON). During activation, each neuron performs a biased sum of the inputs from the neurons to which it is linked. If the entire database exceeds the established limitation amount, the neuron links to additional neurons called transfer [70,71].

**Figure 2:**
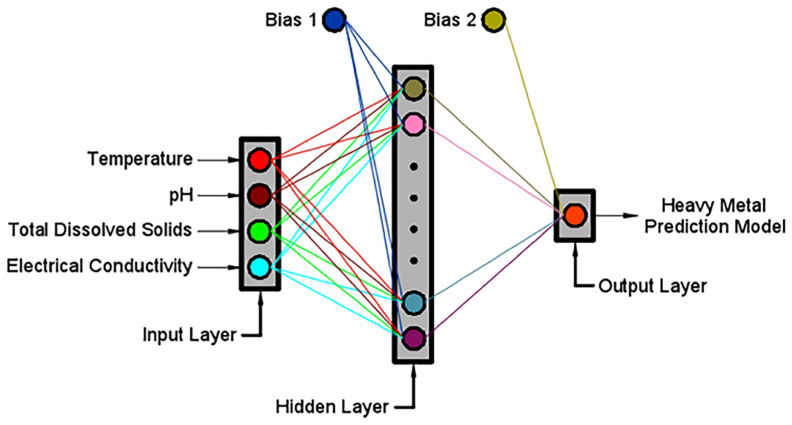
The architecture of the heavy metal prediction models.

The internal characteristics of the surface water and groundwater heavy metal prediction model include the training algorithm and the transfer function. The LM approach (training algorithm) employs a second-order derivative of the performance index but approximates the Hessian matrix using the Jacobian gradient. This approach is the fastest way to train feedforward ANNs with a few hundredweights [72]. For the transfer function, the hyperbolic tangent sigmoid (tansig) is the most often utilized neural activation function for multilayer networks. The tansig functions return outputs scaled between −1 and 1 [73]. The architecture of the heavy metal prediction models is presented in Figure 2.

The complete data array used during the training and testing stages was split into two groups. One set contains 70% of the dataset utilized for network training, whereas the remaining 30% of the data were used for network validation (15%) and testing (15%). The training procedure involved exhibiting entire example pattern pairs in the training dataset to the network and adjusting the weights of the connections until desired values are obtained by the MATLAB Neural Network Toolbox using an iteration-based method. The trained network is exposed to the testing data array to verify the efficiency of the training process after the training procedure is complete [74]. The purpose of the testing step of an ANN model is to ensure that the constructed model was properly trained, and that adequate generalization was achieved. As a result, testing and training the network are basically similar. The testing set is crucial for ensuring that the network has not only remembered a particular dataset but has also learned the application-specific patterns. The testing dataset is a separate dataset that is unknown to the network. After the training phase was completed, the testing dataset is employed to validate and generalize the trained network. When the network can exactly generalize the output for this testing data, the neural network is able to properly forecast the output for new data, and the network is verified [75].

The number of hidden layers was reduced to one, and the number of hidden neurons was limited to 1–30 to prevent an overly complex model. Additionally, the early stopping approach was used to avoid overfitting. The training was halted at the moment of the lowest error [76].

#### 2.3.4. Particle Swarm Optimization

PSO is a well-known evolutionary process that is motivated by the individual interaction and mobility kinetics of insects, birds, and fish. It is a search method based on optimization and population stochastic search that seeks to solve problems in a continuous search space [11]. Particle swarm optimization’s primary benefits over other optimization methods such as ICA and genetic algorithm (GA) are its faster learning rate and lower memory requirements. The particle motions determine the optimal global and optimal personal locations using the PSO. Equations (2) and (3) are the equations for the location and velocity of the particles.

(2) $$X_{n}=X_{c}+V_{n}$$

(3) $$V_{n}=\omega V_{c}+C_{a}r_{a}\left(p_{best}-X_{c}\right)+C_{a}r_{a}\left(g_{best}-X_{c}\right)$$

where *V_c_*, *V_n_*, *X_c_*, and *X_n_* refer to the particle’s current and new velocity and location, respectively. Additionally, *C_a_* and *C_b_* indicate two positive and steady acceleration quantities designated from operators. The variables *r_a_* and *r_b_* refer to arbitrary variables in the form of (0,1), whereas ω denotes the inertia weight (IW) [77].

#### 2.3.5. Hybrid NN-PSO (hN-PSO)

The ANN’s training process generates a minimization issue that may be addressed using either traditional or metaheuristic techniques. In an hH-PSO model, the PSO is used to reduce the ANN’s errors by establishing the model’s optimal weights and biases [78]. The parameters in this study are the weights and biases, and the model’s viable area is based on the interval over which these parameters fluctuate. Equation (4) may be utilized to determine the fitness function of the *i*th particle.

(4) $$E\left(w_{i},b_{i}\right)=\sqrt{\frac{1}{S}\sum_{k=1}^{S}\left[\sum_{l=1}^{O}{\left\{T_{kl}-P_{kl}\left(w_{i},b_{i}\right)\right\}}^{2}\right]}$$

where *E* is the fitness variable, *T_kl_* is the target variable, *P_kl_* is the forecasted variable output based on weights and biases, *S* is the population of training samples, and *O* is the number of neurons.

**Figure 3:**
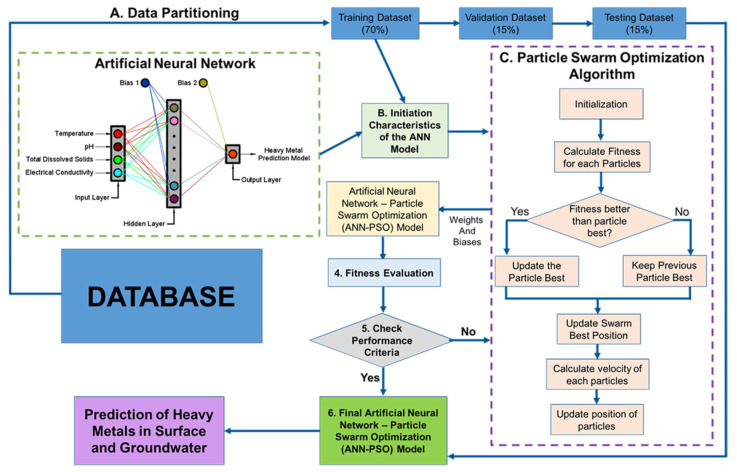
The hN-PSO system.

The following steps are required to implement the hN-PSO model: (a) developing an ANN model with initial weights and biases using a specified number of hidden neurons (HN) in the HL, (b) revising the weights and biases to represent the location of a particle in the model’s “x”-dimensional space, where “x” is the total number of weights and biases, (c) using each particle in each iteration, output values can be predicted and the fitness function value can be calculated using Equation (3), and (d) updating the location of the particles by the PSO algorithm for a specified number of populations and iterations until the target is achieved (fitness function is minimized) [35]. The number of HN for HM models assessed in this research ranges from 1 to 30, as suggested by Tufaner and Demicri in 2020 [79]. Moreover, the number of iterations has been set to 2000 as suggested in the study of Rukhaiyar et al. in 2018 [80]. The hN-PSO system for predicting the heavy metal concentration is shown in Figure 3 [32].

**Figure 4:**
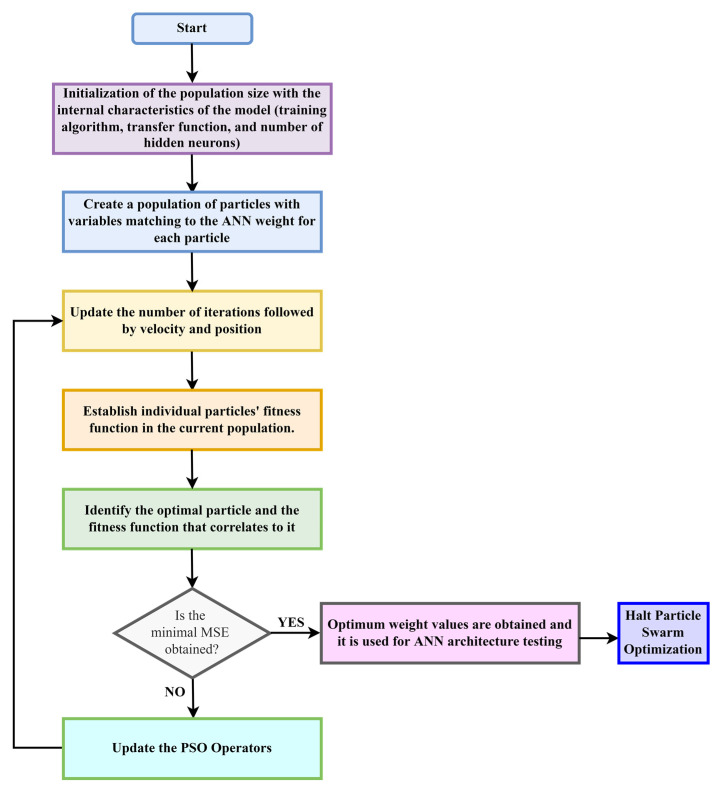
Block diagram of ANN weights optimization using PSO.

PSO was used to train and optimize the initial ANN model. The neural network’s weights and biases were introduced and optimized using the following steps: (a) data collection, (b) network development, (c) network design, (d) initial weights and biases, (e) ANN training using PSO, (f) network validation, and (g) network acceptance as the governing model. Following the preparation of the obtained data, the PSO initialized the particles randomly, including the number of hidden neurons and transfer function and the location and velocity of each particle, which are updated with each iteration. The process starts by traversing the hyperspace of possible solutions in search of the ideal solution. At each iteration, the particles respond adequately depending on their knowledge of other particles. The ANN optimization block diagram using PSO is presented in Figure 4 [81].

#### 2.3.6. Performance Validation and Measurement

Internal and external validation will be used to assess the network’s performance. Internal validation is a component of the model’s development phase and will be carried out in the manner proposed by Thio et al. [82]. An external validation, which includes an external dataset, will be performed to assess the generalizability of the governing neural network architecture when applied to an external set of data [83,84]. The performance measurement criteria used is the Pearson’s correlation coefficient (PCC) and MSE wherein the ideal value is 1 and 0 for the Pearson’s correlation coefficient and MSE, respectively [77,85]. These values are statistical measurement factors employed to compute the connection and the variance concerning computed and forecasted quantities, respectively. Equations for computing the MSE and Pearson’s correlation coefficient are presented in Equations (5) and (6).

(5) $$MSE=\frac{\sum_{i=1}^{n}{\left(O_{i}-P_{i}\right)}^{2}}{n}$$

(6) $$r=\frac{\sum_{i=1}^{n}\left(P_{i}-\bar{P_{i}}\right)\left(O_{i}-\bar{O_{i}}\right)}{\sqrt{\sum_{i=1}^{n}{\left(P_{i}-\bar{P_{i}}\right)}^{2}}\sqrt{\sum_{i=1}^{n}{\left(O_{i}-\bar{O_{i}}\right)}^{2}}}$$

where *n* is the overall quantity of the dataset, and *P_i_* and *O_i_* are the HM concentrations forecasted by the ANN techniques and observed quantities, respectively, whereas the $\bar{O_{i}}$ and $\bar{P_{i}}$ were the average observed values and average forecasted values of the HM concentrations.

Moreover, to extend the evaluation of the models, the Akaike information criterion (AIC) and the Kling–Gupta efficiency (KGE) were also used as performance metrics in this study. The AIC is a measure of goodness and a tool for model selection wherein the least value is the criterion for selecting the best model. At the same time, the KGE is based on the correlation, variability bias, and mean inclination of the model [86,87]. The equations for obtaining the *AIC* and *KGE* are presented in Equations (7) and (8).

(7) AIC=Nln(MSE)+2k

(8) $$KGE=1-\sqrt{{\left(R-1\right)}^{2}+{\left(VE-1\right)}^{2}+{\left(BT-1\right)}^{2}}$$

where *N* is the number of datasets, k is the quantity of HN, R is the linear correlation between the actual and predicted value, *VE* is the variability error, which is the ratio between the standard deviation of the expected and the observed value, and *BT* is the bias term, which is the ratio of the mean predicted and mean actual value. The ideal value for *KGE* is equal to 1.

The optimal architecture of the models, which includes the number of hidden neurons in the hidden layer, was obtained using the MSE and AIC values. The MSE values were utilized to calculate the AIC values for each model topology (hidden neurons from 1 to 30) for SW and GW during the DS and WS. The hidden neuron that corresponds to the minimum AIC values was the governing model structure adopted in each HM model.

### 2.4. Comparison to Other Models

The performance of the developed hN-PSO model for predicting heavy metals in SW and GW were compared to the performance of the other prediction models such as linear and support vector machine models. Linear models include robust linear regression, linear regression, and stepwise linear regression models. Support vector machine models include linear SVM, fine Gaussian SVM, cubic SVM, medium Gaussian SVM, and quadratic SVM.

Robust regression is an effective method for regression analysis and a viable alternative to least squares regression approaches for datasets polluted by outliers or influential observations [88]. MLR determines the correlation between two or more input variables by applying a linear equation to observed data. MLR involves both data summarization and investigation of the connection between variables. The general form of MLR models is presented in Equation (9) wherein *Y* is the independent variable, *X* is the dependent variable, and a_i_ are the regression coefficients [89], and a stepwise regression is a technique for testing the statistical significance of each independent variable in a linear regression model sequentially [90].

(9) $$Y=\sum_{i=1}^{n}a_{i}X_{i}+a_{0}$$

Linear SVM is utilized for linearly separable data, which implies that if a dataset can be categorized into two classes using a single straight line, the data are linearly separable, and a linear SVM classifier is employed [91]. There are three Gaussian SVMs employed in the model comparison, including the fine Gaussian SVM, medium Gaussian SVM, and coarse Gaussian SVM. Fine Gaussian SVM uses a moderate amount of memory for binary classification and a large amount of memory for multiclass classification during the training phase, whereas medium Gaussian SVM uses a moderate amount of memory for binary classification and a large amount of memory for multiclass classification during the training phase. Additionally, the memory consumption of a medium Gaussian SVM is high for multiclass classification and low for binary classification [92]. Coarse Gaussian SVM is a non-linear SVM learning approach that falls under the category of data mining. Furthermore, the coarse Gaussian SVM is difficult to comprehend and has limited flexibility [93]. Additionally, cubic and quadratic SVMs were also compared to the developed NN-PSO models. A cubic SVM is an effective SVM approach when dealing with a memory space constraint wherein SVM locates a hyperplane in a multidimensional space that best separates the classes [94], whereas in quadratic SVM, memory utilization is low for binary classification and high for multiclass classification during its training phase. The prediction speed is likewise rapid for binary classification and slow for multiclass classification [95].

### 2.5. Sensitivity Analysis

Due to the efficiency of the simulation results, the connection weight (CW) segmentation approach was able to calculate the contribution of each input variable to the HM concentration using a sensitivity analysis. Sensitivity analysis was utilized to determine the rate of change in model output as a function of model parameter changes and, as a result, to identify the most influential factors in HM concentrations in surface and groundwater [96,97]. The connections between the neurons are represented by the CW, which means the connections between the issue and the resolution. Sensitivity analysis was used to establish the RI of individual input parameters. Olden’s CW technique was used in this work to estimate the variable significance of individual input variables to the number of heavy metals in SW and GW [98].

The CW approach was used to compute the product of the IN–HN and HN–ON, CWs for each IN and ON, and then add the products across the HN. The higher the sum, the more significant the IN is. Equation (10) is used to compute the RI of an input variable “*i*”.

(10) $$R.I._{i}=\frac{\sum_{x=1}^{w}W_{ix}W_{xy}}{\sum_{i=1}^{z}\sum_{x=1}^{w}W_{ix}W_{xy}}\times 100$$

where *R.I._i_* is the variable significance of the variable “*i*” in the input layer (IL) to the HM concentration, *x* is the index quantity of the HN, *W_ix_* is the CW between the input parameter “*i*” and the HN noted as *x*, and *W_xy_* is the CW between the HN noted as *x* and the ON noted as *y* [99].

## 3. Results

This section summarizes the principal results and includes all models developed using neural networks coupled with particle swarm optimization technique. This section also provides the result of the sensitivity analysis of the variables and their influence on the HM concentrations in SW and GW as well as uncertainty analysis of the HM models.

### 3.1. Heavy Metal Concentrations

The dataset for the physicochemical characteristics and HM concentrations used in the study are depicted in Appendix B. The physicochemical characteristics observed include temperature (Figure A1), pH (Figure A2), EC (Figure A3), and TDS (Figure A4). The heavy metal concentrations observed include Cr (Figure A5), Cd (Figure A6), Fe (Figure A7), Mn (Figure A8), Zn (Figure A9), Ni (Figure A10), Pb (Figure A11), and Cu (Figure A12).

Table 3 and Table 4 enumerate descriptive information on the HM concentrations in SW samples from Marinduque Province. The mean surface water temperature for both the dry and wet seasons was within the Philippine Water Quality Guidelines (WQG). The mean surface water pH for both the dry and wet seasons was less than the minimum pH value set by the Philippine WQG and the WHO standards. The mean EC and TDS of surface water were greater than the guidelines set by the WHO, which are 1500 µS/cm and 1200 mg/L, respectively. The mean concentration of total Cd, Fe, and Cu was above the Philippine WQG and the World Health Organization guidelines (WHO). The mean concentration of Cr in SW was above the Philippine WQG. Ni concentration also exceeded the guidelines set by the WHO for both dry and wet seasons.

The GW physicochemical characteristics recorded were below the Philippine National Standards for Drinking Water (PNSDW) 2017 and WHO requirements for both dry and rainy seasons. Likewise, the EC and TDS were within the World Health Organization limits. The total mean concentration of Cr, Cd, Fe, Zn, and Ni exceeded the PNSDW 2017 and the WHO guidelines. Table 5 and Table 6 include descriptive data for the physicochemical parameters and total concentrations of HM utilized in the DS and WS-GW models.

### 3.2. Correlation Analysis

**Figure 5:**
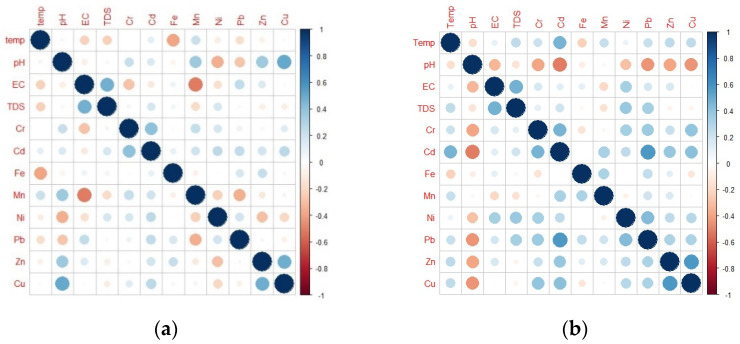
Pearson’s correlation matrix plots for the surface water physicochemical parameters and HM concentrations during (**a**) DS; and (**b**) WS.

The PCC was applied to examine the degree of association between physicochemical properties and measured HM concentrations in SW throughout the dry and rainy seasons. Pearson’s correlation matrix (PCM) plots of these parameters across the DS and WS are shown in Figure 5. It was discovered that during both the DS and WS, the EC and TDS, as well as Cr and Cd, were positively associated. This recorded data confirmed the study of Tiwari et al. [101] and Huang et al. [102]. Cu and Zn also exhibited a positive association throughout the dry and wet seasons, agreeing with the findings of Bhuyan et al. [103]. During the wet season, a positive relation between Cd:Cu and Cd:Pb, as well as Ni and Pb, was recorded. These data agreed with the findings of Wang et al. [104]. These positive correlations among the heavy metals could indicate that the water body in the research area has similar hydro-chemical features [105].

**Figure 6:**
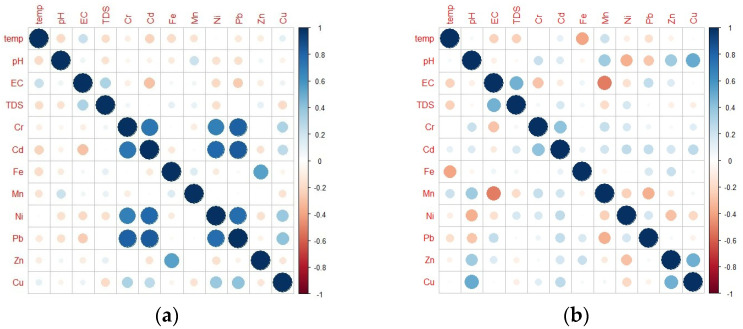
Pearson’s correlation matrix plots for the groundwater physicochemical parameters and HM concentrations during (**a**) DS; and (**b**) WS.

Correlations between physicochemical characteristics and heavy metals in groundwater were also studied. It was shown that EC and TDS had a positive relationship for both dry and wet seasons as illustrated by Figure 6. The positive association between EC and TDS is due to conductivity being a function of total dissolved solids, the ionic composition of water, and the concentration of dissolved species [106]. Likewise, a positive correlation was observed for Cd and Cr for both the dry and wet seasons, which suggests a common origin of these heavy metals [107]. Additionally, it was established that during the dry season, Cr has a positive link with Ni and Pb, which was confirmed by the results of Ukah et al. and Magesh et al. [105,108]. Moreover, similar to the findings of Rashid et al. in 2021 [109], there was a high positive correlation between Cd:Ni and Cd:Pb during the WS. Furthermore, a positive correlation was recorded between Fe and Zn. This is consistent with Senoro et al. results in 2022 [110]. Positive correlations between these metals showed that they originated from a common source. The loadings from Cd, Pb, and Ni were associated to anthropogenic sources as observed by Wagh et al. in 2018 [111]. The Fe and Zn correlation was geogenic in nature as elaborated in the findings of Bhutiani et al. in 2016 [112].

### 3.3. NN-PSO Modelling Results

Table 7 and Table 8 illustrate the NN-PSO simulations for the HM models in SW during the dry and rainy seasons, respectively. This section contains the R and MSE findings, as well as the topology of the created HM models. The topology of the heavy metal models was described as 4-HN-1 (input neurons-hidden neurons-output neurons), wherein the number of hidden neurons for each HM model is likewise shown in Table 7 and Table 8.

The R validation of HM models in SW varied between 0.95566 and 0.99972 during the DS and between 0.95686 to 0.98779 during the WS. The R values varied from 0.95710 to 0.98620 and 0.94923 to 0.98897, respectively, during the DS and WS throughout the testing phase. The highest MSE was observed in the Cu model, whereas the lowest MSE was observed in the Cr model for SW during DS. Moreover, the highest MSE was observed in the Fe model, whereas the lowest MSE was observed in the Mn model in the SW for the period of the WS.

The R validation of HM models in GW varied between 0.97786 and 0.99965 during the dry season and between 0.96040 to 0.99925 during the wet season. The R values varied from 0.95538 to 0.99835 and 0.97544 to 0.99815, respectively, during the DS and WS throughout the testing phase. The Mn model had the greatest MSE, whereas the Pb model had the lowest for GW during DS. Additionally, the Fe model had the greatest MSE, whereas the Cu model had the lowest MSE in GW during the WS. The NN-PSO simulation findings for the HM models in GW during the DS and WS are shown in Table 9 and Table 10.

The validation and testing plots of the SW and GW during the DS and WS are exhibited in Appendix C. These include the plots for Cr (Figure A13), Cd (Figure A14), Fe (Figure A15), Mn (Figure A16), Zn (Figure A17), Ni (Figure A18), Pb (Figure A19), and Cu (Figure A20).

The optimal network parameters of the developed HM models (IN-HN-ON) for SW and GW are presented in Table 11. These include the training algorithm used (Levenberg–Marquardt algorithm), transfer function (tansig), and the number of hidden neurons for each model of SW and GW during the dry and wet seasons.

**Figure 7:**
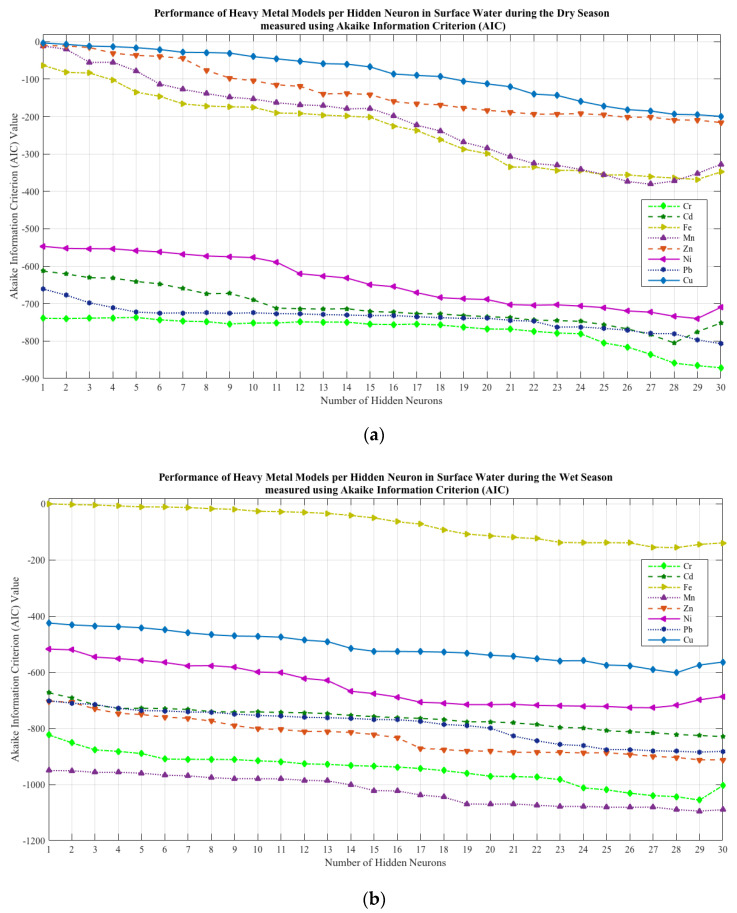

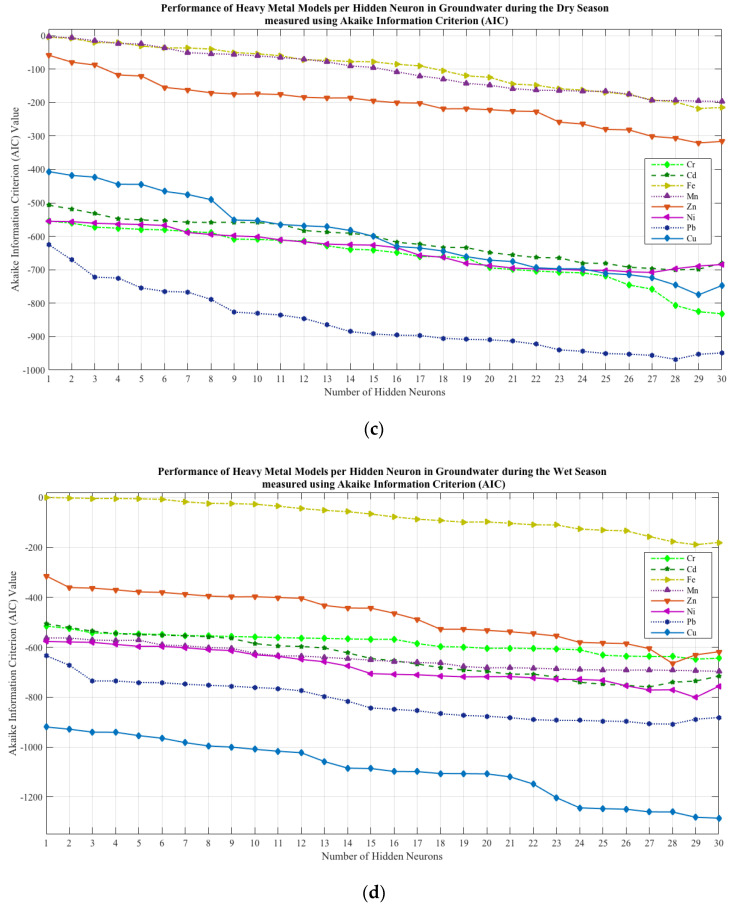
Effect of the hidden neurons on the heavy metal model performance measured using AIC: (**a**) surface water—dry season; (**b**) surface water—wet season; (**c**) groundwater—dry season; (**d**) groundwater—wet season.

Figure 7 illustrates the link between the quantity of HN varying from 1 to 30 and the corresponding AIC values. It was observed that the lowest AIC values were seen in 27 HN for Mn (SW dry), Ni (SW wet and GW dry), and Cd (GW wet); 28HN for Cd (SW dry and GW dry), Fe (SW wet), Cu (SW wet), Pb (GW dry and GW wet), and Zn (GW wet); 29 HN for Fe (SW dry, GW dry, and GW wet), Ni (SW dry and GW wet), Cr (SW wet and GW wet), Mn (SW wet), Pb (SW wet), Zn (GW dry), and Cu (GW dry); and 30 HN for Cr (SW dry and GW dry), Zn (SW dry and SW wet), Pb (SW dry), Cu (SW dry and GW wet), Cd (SW wet), and Mn (GW dry and GW wet). These lowest AIC values observed in the respective hidden neurons were included in the metrics used to identify the governing model for each target HM.

**Figure 8:**
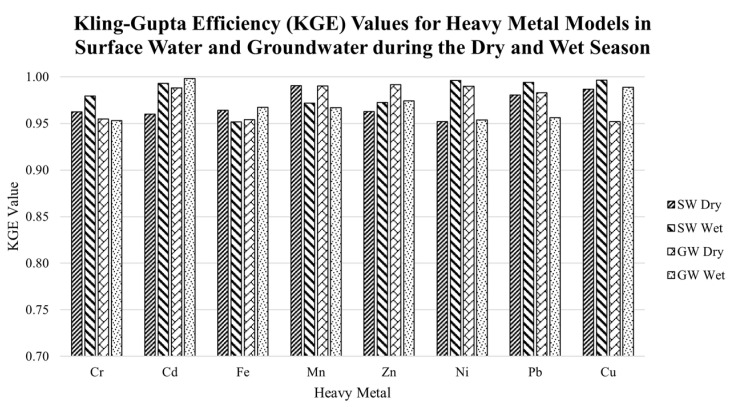
KGE values for SW and GW Models during the dry and wet season.

The results of the additional evaluation metric using the KGE are presented in Figure 8. Results show that the developed models for the SW and GW during the dry and wet seasons have good KGE values, wherein all values were greater than 0.95.

### 3.4. Comparison to Other Models

**Figure 9:**
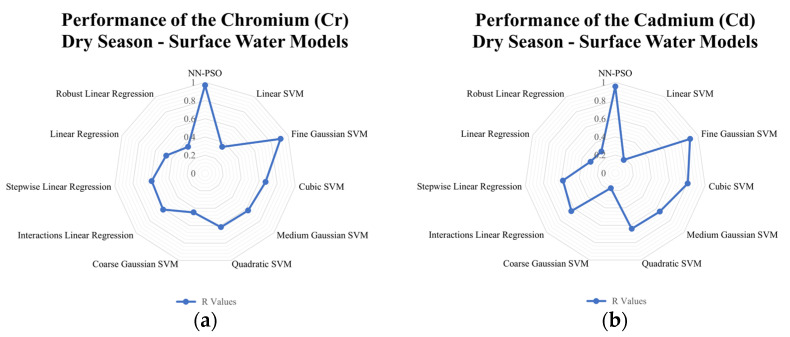

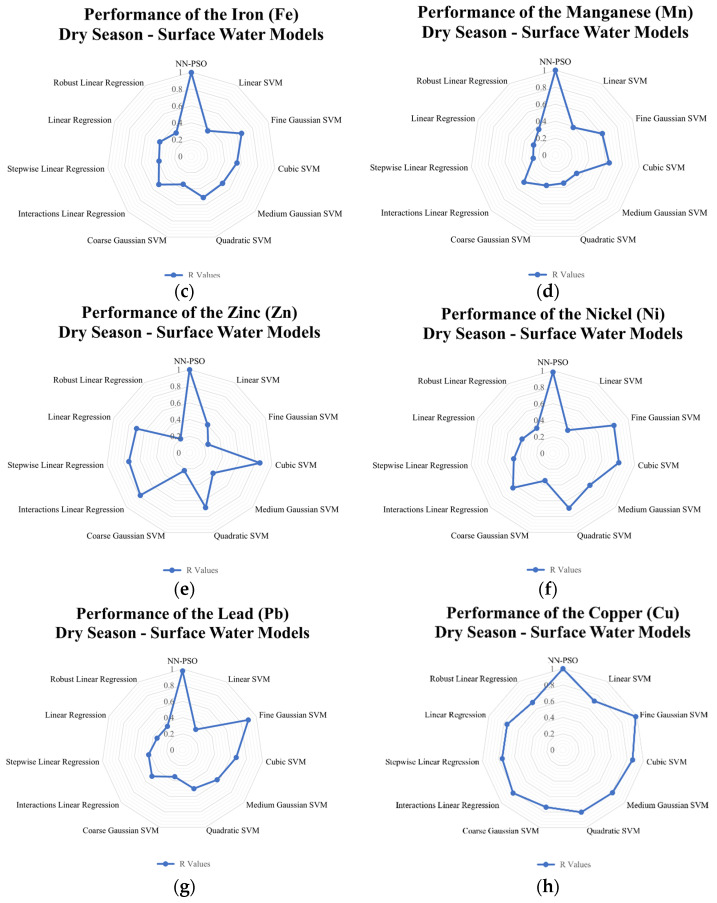
Radar graph showing the performance of the (**a**) Cr; (**b**) Cd; (**c**) Fe; (**d**) Mn; (**e**) Zn; (**f**) Ni; (**g**) Pb; (**h**) Cu surface water models during the dry season.

**Figure 10:**
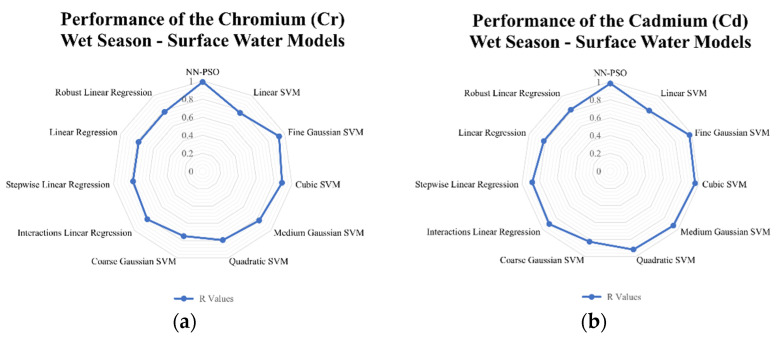

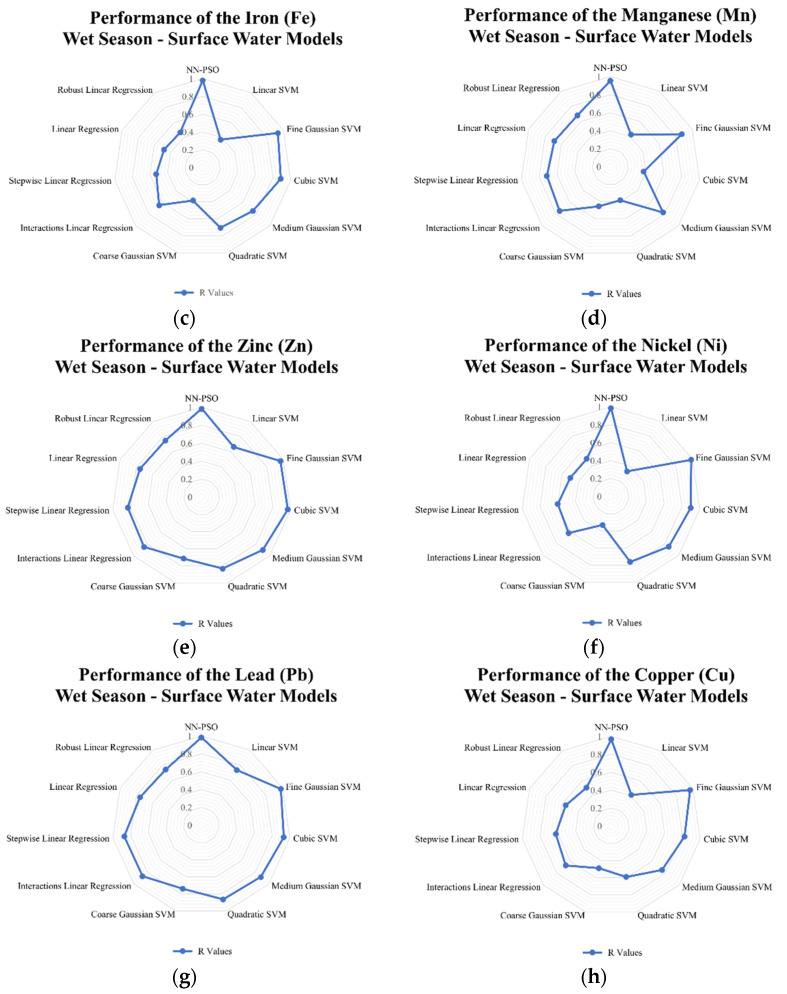
Radar graph showing the performance of the (**a**) Cr; (**b**) Cd; (**c**) Fe; (**d**) Mn; (**e**) Zn; (**f**) Ni; (**g**) Pb; (**h**) Cu surface water models during the wet season.

**Figure 11:**
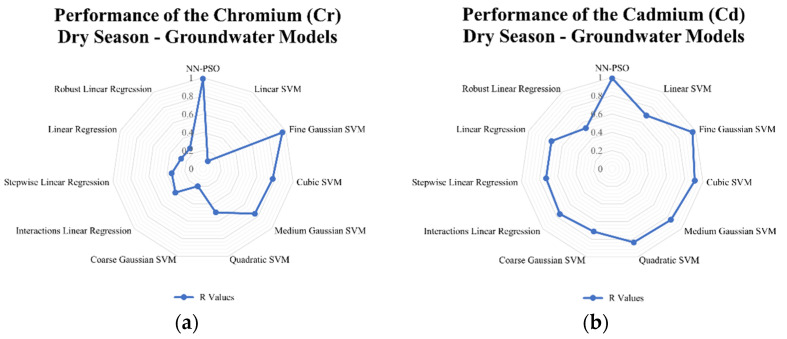

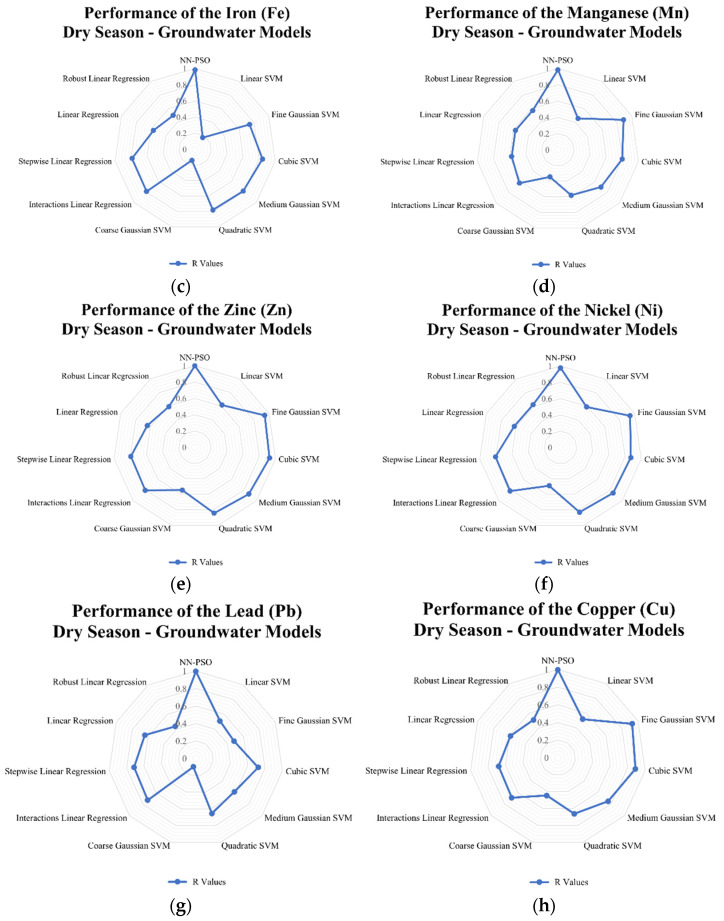
Radar graph showing the performance of the (**a**) Cr; (**b**) Cd; (**c**) Fe; (**d**) Mn; (**e**) Zn; (**f**) Ni; (**g**) Pb; (**h**) Cu groundwater models during the dry season.

**Figure 12:**
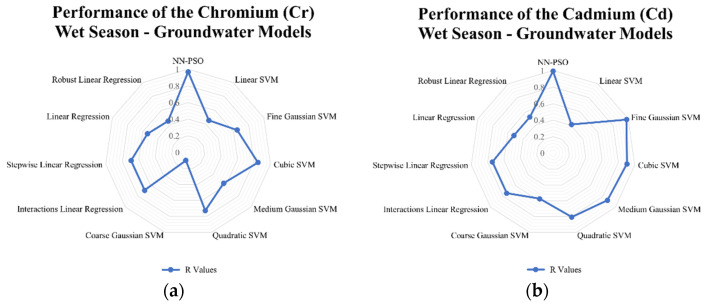

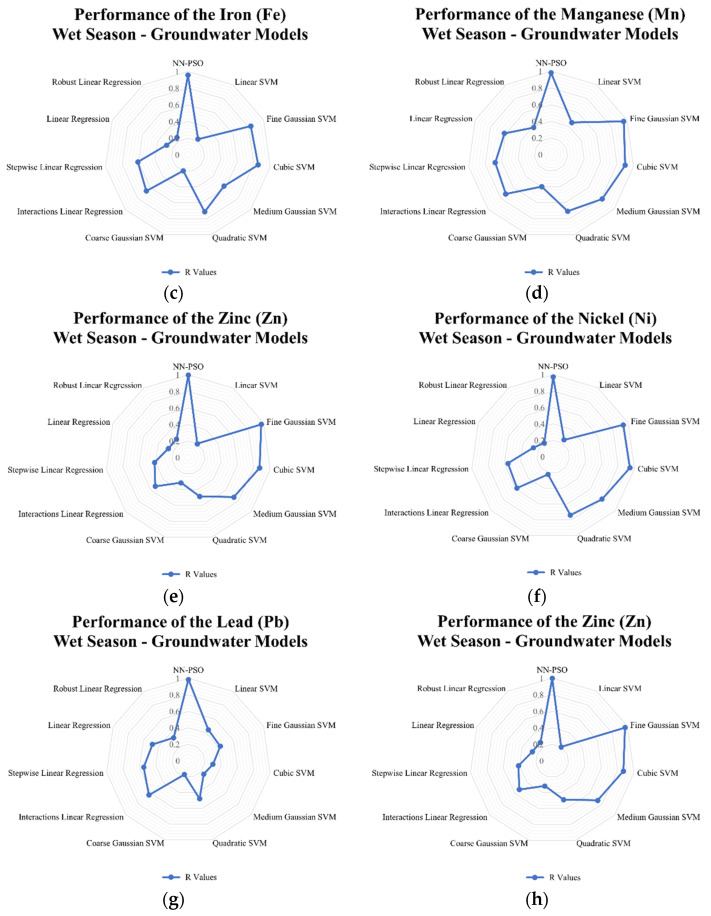
Radar graph showing the performance of the (**a**) Cr; (**b**) Cd; (**c**) Fe; (**d**) Mn; (**e**) Zn; (**f**) Ni; (**g**) Pb; (**h**) Cu groundwater models during the wet season.

Linear and support vector machine models were likewise developed to see how well the developed hybrid NN-PSO models performed compared to other models. The results indicated that for surface water models generated using NN-PSO, the R-values were up to 1.60 times more than the R-values obtained using the SVM models and 1.2 to 2.0 times greater than the R-values obtained using the linear models during the dry season. For the wet season surface water model, the R values for HM models utilizing NN-PSO were up to 1.1 times and 1.6 times greater than the highest R-value observed from the SVM models and linear models, respectively. Similarly, the R values obtained from the heavy metal dry season groundwater models were up to 1.4 times greater than the highest R-value obtained from the SVM models and 1.2 to 2.5 times greater than the R-values obtained from the linear models. Moreover, wet season ground-water models were shown to have R-values up to 2.1 times and 1.9 times greater than the highest recorded R-value for models created using SVM and linear models, respectively. The radar graphs of the observed values for the generated NN-PSO models and the linear and SVM models are shown in Figure 9, Figure 10, Figure 11 and Figure 12.

### 3.5. Sensitivity Analysis Using Olden’s Connection Weight Approach

**Figure 13:**
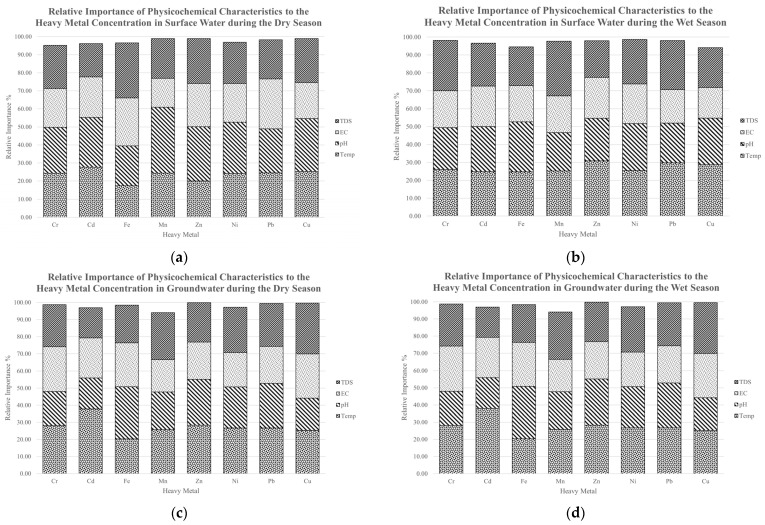
The relative importance of the physicochemical parameters to the heavy metal concentration in (**a**) SW during the DS; (**b**) SW during the WS; (**c**) GW during the DS; (**d**) GW during the DS.

The CW of the models for DS and WS were utilized to calculate the RI of the physicochemical parameters to the HM concentrations. Using Olden’s CWs approach, the results of the calculation of R.I. in each model are shown in Figure 13.

It was observed in the DS SW models that the surface water pH is the parameter with the highest RI to the Cr, Mn, Zn, Ni, and Cu concentration, whereas that of Cd, Pb, and Fe were seen to be temperature, EC, and TDS, respectively. The SW models during WS displayed temperature to have the highest R.I. to the Zn, Pb, and Cu concentration, whereas that of Cr and Mn was determined to be the TDS. It was also observed that pH is the most influential parameter for the Cd, Fe, and Ni concentrations.

For the GW models during DS, the temperature was observed to have the highest R.I. to the Cr, Cd, Zn, Ni, and Pb concentration, whereas TDS recorded the highest RI for Mn and Cu. It was likewise seen that groundwater pH is the most influential factor for the Fe concentration in the DS. For the WS groundwater models, the temperature was observed to have the highest R.I. for Cr and Cd, whereas it was EC for Fe and TDS for Ni and Cu. It was also observed that the groundwater pH during the wet season is the most significant parameter for the Ni, Zn, and Pb concentrations.

## 4. Discussion

The HM contaminants in SW and GW endanger human life and contribute to the deterioration of the environment and health risks. Detected HM concentrations in SW and GW were higher than the WHO and Philippine standards. As water is a vital part of everyday activity of a community such as Marinduque Province, guaranteeing universal access and sustainable management of water should be ensured as mandated by the United Nations Sustainable Development Goals (SGDs). Accurately predicting HM concentration in water resources is significant to ensure that proper monitoring of its progression in the environment is implemented. It also enables the proper dissemination of information of the potential risks that a community might be exposed to and creates mitigating measures and remediation strategies that the authorities could implement to address the heavy metal contamination in water resources. The ML models are fundamentally data-driven, with several studies implementing different ML techniques with different variables used as the input parameters. The ANN-PSO technique was implemented in this study to develop the HM intensity models. The developed NN-PSO models were compared to models created using linear and SVM methods. It was observed that the developed NN-PSO models for heavy metals in surface water during the dry and wet season performed better than the models created using linear and SVM models. The observed R-values were up to 1.6 times and 1.1 times greater than the highest R-values observed for linear and SVM models during the dry and wet seasons. Moreover, the GW models were observed to have R-values that were greater than up to 1.4 times and 1.6 times the R-values observed in the highest linear and SVM models during the DS and WS. The findings were in agreement with the results of the study by Zhang et al. in 2020 wherein the hybrid ANN-PSO was observed to perform better as compared to the other MLR and machine learning models including SVM [32].

**Figure 14:**
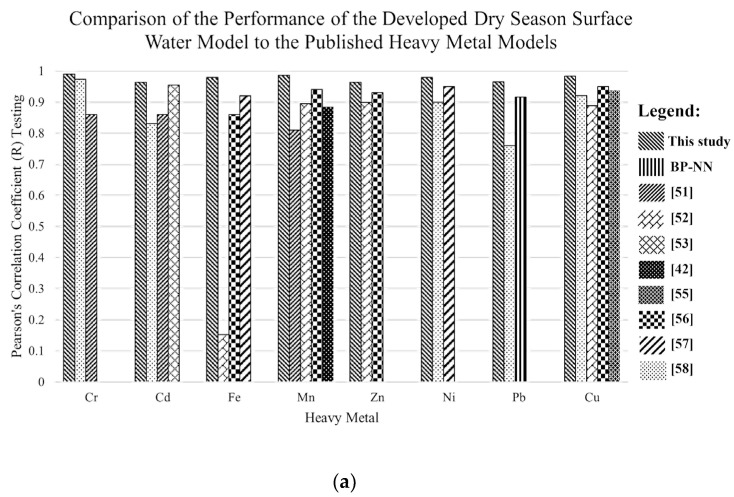

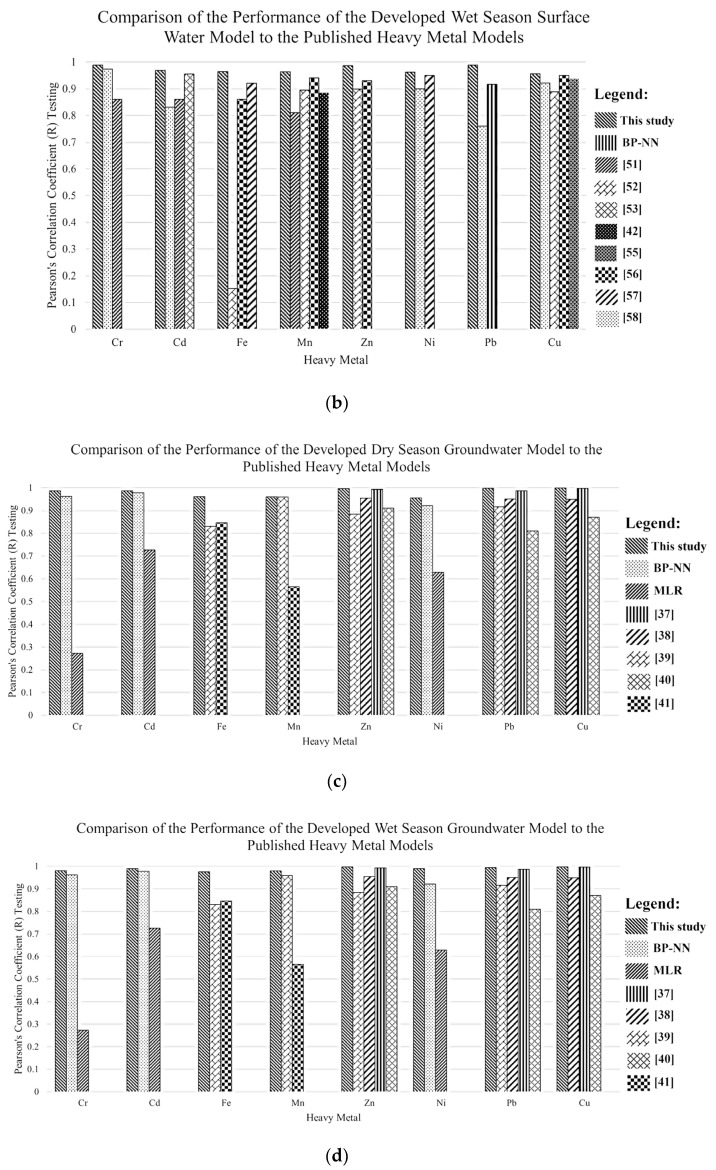
Comparison of the performance of the published models and the developed models for (**a**) DS surface water; (**b**) WS surface water; (**c**) DS groundwater; (**d**) WS groundwater.

Furthermore, comparisons in the performance of the developed models to the previously published models are shown in Figure 14. These models include different ML techniques such as NN-PSO, NN-BR, NN-ICA, NN-LM, NN-BBO, MANFIS-SCM, SVM, MANFIS-GP, MANFIS-SCM, ANFIS, K-NN, and GRNN. In this study, a hybrid ANN-PSO model, i.e., hN-PSO, was used with input parameters that were easier to obtain. The findings reveal that the present study’s performance measures are on par with the performance of previously published heavy metal models, with the additional advantage of using parameters that can be collected in-situ.

The simulation of different internal characteristics and topologies of models were implemented to obtain the governing models of HMs total concentration in SW and GW. It was observed that the governing models were based on the AIC performance metric values, which are the minimum among the observed HN. It was observed that as the AIC value reached its minimum value, further increasing the number of HN will return an increased AIC value. This implies that the network has already become generalized [113]. In addition, KGE values for all models in surface and groundwater during the dry and wet seasons were greater than 0.95, which suggests that the models are accurate. Additionally, it was discovered that the quantity of HNs had no influence on the model’s performance, which is consistent with Çolak’s findings in 2021 [114].

Olden’s CW technique is an excellent strategy for illuminating the neural network’s black box design by offering more explanatory insight into the input parameters’ contributions [115]. The Olden’s CWs approach was implemented in this study because it was the best method as suggested in the findings of Olden et al. [116]. In this study, it was observed that the temperature and pH are the most influential input parameters to the HM concentrations; these findings were also observed in Morin and Mutt [117].

The use of ML tools such as NN-PSO models is in line with the transition of different disciplines to Industry 4.0. Employing ML techniques provides a new avenue to create models that can be applied in real-world conditions that can be complex and non-linear in nature. The PSO method was used with the ANN model in this research to create the optimal model with the least error.

## 5. Conclusions

Limited HM concentrations data in SW and GW inspired the researchers to develop models as tools for environmental quality monitoring specifically on the total concentration of HMs such as Cr, Cd, Fe, Mn, Zn, Ni, Pb, and Cu. Using the physicochemical properties of SW and GW, which are the most commonly monitored data, the NN-PSO models were developed and exhibited good performance that was on par with linear, SVM, and existing published deterministic and AI models that used input parameters that cannot be obtained in field conditions. The performance of the models was evaluated using their AIC values, and the number of HN that returned the lowest AIC value was chosen as the governing topology for the HM models. It was further observed that as the AIC value reached its minimum value, further increasing the number of HN returned an increased AIC value due to network generalization. Moreover, the R and KGE values of the governing model were almost equal to 1, whereas MSE values were approaching 0, which are the ideal values for these performance metrics. The performance of the developed NN-PSO models was compared to created models using linear and SVM approaches, and the findings suggest that the NN-PSO was the superior modeling tool in this study based on its model performance. The R.I. of the input parameters was assessed using Olden’s CWs approach. The sensitivity analysis showed that temperature and pH were the parameters with the most influence on the HM concentration.

Based on the elaborated findings, it can be concluded that the use of the hN-PSO for forecasting HM concentrations in water resources is both an effective and a practical approach. Additionally, the recorded findings indicate the efficacy of using common water parameters as inputs to prediction models for HM concentrations that may be simply adopted for in-situ settings. These findings could initiate and contribute to regular monitoring of HM concentrations in SW and GW.

The findings of the study illustrate that the NN-PSO is an effective method and practical approach in predicting HM concentration in water resources. Moreover, this study provides a model with parameters suitable for in-situ purposes and on par with the other HM prediction models in water resources. Future studies on the inclusion of HM speciation and its prediction models with more data dimensions, or the use of feature reduction approach in model development are recommended. Multi-layered networks using deep learning and other ML may also be studied. Integration of time components is another facet for future study that will be investigated using time series-based methods such as NARX and LSTM, which can be used in dynamic analysis and transport studies.

## Figures and Tables

**Table 1:** List of abbreviations and symbols used in this study.

Abbreviation/ Symbol	Description	Abbreviation/ Symbol	Description
AAS	Atomic Absorption Spectroscopy	IoT	Internet of Things
AI	Artificial Intelligence	KGE	Kling-Gupta Efficiency
AIC	Akaike Information Criterion	KNN	K-Nearest Neighbor
AMD	Acid Mine Drainage	LM	Levenberg-Marquardt
ANN	Artificial Neural Network	LSTM	Long Short-Term Memory
BBO	Biogeography-Based Optimization	LSW	Lake Surface Water
BP	Back Propagation	M5P	Model Tree
BR	Bayesian Regularization	MANFIS	Multi-output Adaptive Neuro-Fuzzy Inference System
CA	Cluster Analysis	MHMI	Modified Heavy Metal Index
CI	Contamination Index	ML	Machine Learning
CSW	Coastal Surface Water	MLGI	Machine Learning Geostatistical Interpolation
EBK	Empirical Bayesian Kriging	MLR	Multiple Linear Regression
EHCI	Entropy Weight-based HM Conc. Index	NARX	Non-linear AutoRegressive eXogeneous
FCM	Fuzzy c-means Clustering Method	PLI	Pollution Load Index
GA	Genetic Algorithm	PMI	Principal Component Analysis-based Metal Index
GEP	Gene Expression Programming	PSO	Particle Swarm Optimization
GFF	Generalized Feed Forward	RBF	Radial Basis Function
GP	Grid Partitioning	RI	Relative Importance
GRNN	Generalized Regression Neural Network	SCM	Subtractive Clustering Method
HEI	Heavy Metal Evaluation Index	SPI	Synthetic Pollution Index
hN-PSO	Hybrid Neuro-Particle Swarm Optimization	SVM	Support Vector Machine
HPI	Heavy Metal Pollution Index	SVM-Poly	SVM with Polynomial
ICA	Imperialist Competitive Algorithm	WQG	Water Quality Guidelines
ICP-OES	Inductively Coupled Plasma-Optical Emission Spectrometry	WQI	Water Quality Index

**Table 2:** Published heavy metal prediction models in groundwater and surface water.

Prediction Method	Sample Type	Target Output(s) of the Model	Reference
ANN–PSO, ANN- Bayesian Regularization (BR)	GW	As, Cu, Pb, Zn	[37]
ANN–Imperialist Competitive Algorithm (ICA), ANN–Levenberg–Marquardt (LM)	GW	As, Cu, Pb, Zn	[38]
ANN, ANN–Biogeography–Based Optimization (BBO) Algorithm, Multi-output Adaptive Neuro-Fuzzy Inference System (MANFIS)–Subtractive Clustering Method (SCM)	GW	Fe, Mn, Pb, Zn	[39]
SVM based Regression–Radial Basis Function (RBF)	GW	Pb, Zn, Cu	[40]
ANN	GW	Si, Al, Fe, K, Ca, Na, Mg, Cl, Mn, Sr, Br(Groundwater)	[41]
BP-NN, Nonlinear AutoRegressive eXogenous (NARX)	GW	As	[42]
ANN	GW	Water Quality Index	[43]
ANN, MLR	GW	pH, EC, TDS, TH, MHMI, PLI, SPI	[44]
ANN, Deep Learning	GW	HPI, HEI, CI, EHCI, HMI, PMI	[45]
MLP-NN, Elman-NN, GFF-NN	GW	Pb, Zn, As	[46]
BP-NN	GW	Turbidity, Fe, Cl, SO_4_, TDS, TH, Mn, Zn, KMnO_4_ Index, NO_3_-N, NO_2_-N, NH_3_-N, F	[47]
MLR, BP-NN, GEP	SW	WQI	[48]
NARX, BP-NN	CSW	Cr, Ni, Cu, Pb	[49]
K-Means CA, BP-NN	LSW	Fe, Cu	[50]
ANN, SVM	SW	Ti, Cu, Mn, Ni, As, Cd, Sb, Pb	[51]
MANFIS–Grid Partitioning (GP), MANFIS-SCM, MANFIS–Fuzzy c-means Clustering Method (FCM)	SW	Cu, Fe, Mn, Zn	[52]
Adaptive Neuro–Fuzzy Inference System (ANFIS)	SW	Cd	[53]
ANN–LM, ANN–ICA	SW	As, Cu, Pb, Zn	[38]
ANN	SW	Mn	[42]
ANN, SVM with Polynomial (SVM-Poly), SVM–RBF, Model Tree (M5P), K–Nearest Neighbor (K-NN)	SW	Cu	[54]
ANN	SW	Cu	[55]
SVM, Generalized Regression Neural Network (GRNN)	SW	Cu, Fe, Mn, Zn	[56]
SVM, ANN	SW	Ni, Fe	[57]
BP-LM	SW	Cd, Cr, Cu,	[58]

**Table 3:** Descriptive statistics of the physicochemical parameters and total concentration of HM (in mg/L) used in the DS–SW model.

Parameter	N	Min	Max	Mean	Guidelines	
					Philippine WQG [100]	WHO
Temp (°C)	80	26.0	36.4	30.58	25–31	-
pH	80	2.9	9.4	6.28	6.5–9.0	6.5–9.2
EC (µS/cm)	80	130.0	6000.0	2617.21	-	1500
TDS (mg/L)	80	60.0	3000.0	1377.66	-	1200
Cr (mg/L)	80	0.00029	0.03766	0.01820	0.010	0.050
Cd (mg/L)	80	0.00706	0.06122	0.04315	0.005	0.003
Fe (mg/L)	80	0.45237	2.76195	2.32390	1.500	0.300
Mn (mg/L)	80	0.00049	11.09783	2.07269	0.200	0.400
Zn (mg/L)	80	0.00047	9.58050	1.69057	2.000	3.000
Ni (mg/L)	80	0.00413	0.12689	0.10156	0.200	0.070
Pb (mg/L)	80	0.00339	0.05608	0.03851	0.050	0.010
Cu (mg/L)	80	0.02763	17.16567	7.67426	-	2.000

**Table 4:** Descriptive statistics of the physicochemical parameters and total concentration of HM (in mg/L) used in the WS–SW model.

Parameter	N	Min	Max	Mean	Guidelines	
					Philippine WQG [100]	WHO
Temp (°C)	80	26.7	33.7	30.26	25–31	-
pH	80	3.1	8.4	5.94	6.5–9.0	6.5–9.2
EC (µS/cm)	80	90	5380.0	2211.00	-	1500.00
TDS (mg/L)	80	40	2670.0	1142.35	-	1200.00
Cr (mg/L)	80	0.00023	0.03766	0.02937	0.010	0.05
Cd (mg/L)	80	0.00040	0.06122	0.04459	0.005	0.003
Fe (mg/L)	80	0.06915	53.01624	21.74808	1.5	0.3
Mn (mg/L)	80	0.00361	0.01769	0.01027	0.2	0.4
Zn (mg/L)	80	0.02480	0.07430	0.03922	2.00	3.00
Ni (mg/L)	80	0.00415	0.12689	0.08820	0.20	0.07
Pb (mg/L)	80	0.00680	0.05607	0.03458	0.05	0.01
Cu (mg/L)	80	0.00690	0.20730	0.09144	-	2.00

**Table 5:** Descriptive statistics of the physicochemical parameters and total concentration of HM (mg/L) used in the DS–GW model.

Parameter	N	Min	Max	Mean	Guidelines	
					PNSDW 2017 [67]	WHO
Temp (°C)	80	26.3	49.6	37.72	-	-
pH	80	6.1	7.9	7.01	6.5–8.5	6.5–9.2
EC (µS/cm)	80	80.0	2350.0	1140.45	-	1500.000
TDS (mg/L)	80	30.0	1150.0	499.12	600.000	1200.000
Cr (mg/L)	80	0.01733	0.17182	0.07527	0.050	0.050
Cd (mg/L)	80	0.00055	0.10389	0.06879	0.003	0.003
Fe (mg/L)	80	0.00038	54.68567	11.50116	1.000	0.300
Mn (mg/L)	80	0.00009	8.71857	2.44137	0.400	0.400
Zn (mg/L)	80	0.00098	56.96133	13.95211	5.000	3.000
Ni (mg/L)	80	0.00013	0.12530	0.08955	0.070	0.070
Pb (mg/L)	80	0.01560	0.12178	0.10676	0.010	0.010
Cu (mg/L)	80	0.03711	0.26050	0.21542	1.000	2.000

**Table 6:** Descriptive statistics of the physicochemical parameters and total concentration of HM (in mg/L) used in the WS–GW model.

Parameter	N	Min	Max	Mean	Guidelines	
					PNSDW 2017 [67]	WHO
Temp (°C)	80	26.2	36.7	30.25	-	-
pH	80	5.6	7.9	6.85	6.5–8.5	6.5–9.2
EC (µS/cm)	80	20.0	2840.0	1185.05	-	1500.000
TDS (mg/L)	80	10.0	1400.0	601.20	600.00	1200.000
Cr (mg/L)	80	0.01638	0.17179	0.14767	0.050	0.050
Cd (mg/L)	80	0.00055	0.10389	0.04458	0.003	0.003
Fe (mg/L)	80	0.16390	13.58610	9.82432	1.000	0.300
Mn (mg/L)	80	0.00405	0.14579	0.04089	0.400	0.400
Zn (mg/L)	80	0.02480	0.51992	0.26563	5.000	3.000
Ni (mg/L)	80	0.00101	0.12490	0.10005	0.070	0.070
Pb (mg/L)	80	0.05496	0.12178	0.11831	0.010	0.010
Cu (mg/L)	80	0.00690	0.02759	0.02257	1.000	2.000

**Table 7:** NN-PSO simulation results for the heavy metal models in SW during the DS.

	Hidden Neurons	No. of Particles	No. of Iterations	Elapsed Time (sec)	MSE	R	
						Validation	Testing
Cr	30	7	2000	121.2884	0.000009	0.96878	0.98999
Cd	28	3	2000	153.1887	0.000021	0.95566	0.96404
Fe	29	9	2000	150.5006	0.004871	0.99585	0.97976
Mn	27	3	2000	151.0693	0.004364	0.99933	0.98620
Zn	30	6	2000	116.5287	0.031773	0.99904	0.96388
Ni	29	6	2000	115.7815	0.000047	0.98316	0.97981
Pb	30	4	2000	152.8467	0.000020	0.97832	0.96557
Cu	30	2	2000	153.2260	0.039010	0.99972	0.98390

**Table 8:** NN-PSO simulation results for the heavy metal models in SW during the WS.

	Hidden Neurons	No. of Particles	No. of Iterations	Elapsed Time (sec)	MSE	R	
						Validation	Testing
Cr	29	2	2000	157.2246	0.0000009	0.99050	0.98830
Cd	30	8	2000	152.5088	0.0000150	0.98307	0.96868
Fe	28	10	2000	151.0597	0.0702760	0.98099	0.96368
Mn	29	10	2000	138.3126	0.0000006	0.95686	0.96337
Zn	30	3	2000	114.7199	0.000005	0.98559	0.98614
Ni	27	1	2000	120.5753	0.000058	0.98779	0.96227
Pb	29	1	2000	119.6443	0.000008	0.98377	0.98897
Cu	28	9	2000	153.6068	0.000269	0.96707	0.95589

**Table 9:** NN-PSO simulation results for the HM models in GW during the DS.

	Hidden Neurons	No. of Particles	No. of Iterations	Elapsed Time (sec)	MSE	R	
						Validation	Testing
Cr	30	8	2000	154.5653	0.000014	0.98851	0.98640
Cd	28	1	2000	156.7819	0.000078	0.98910	0.98683
Fe	29	9	2000	150.3615	0.031866	0.98414	0.96054
Mn	30	6	2000	158.4757	0.040315	0.98414	0.96002
Zn	29	4	2000	122.3900	0.008780	0.99965	0.99577
Ni	27	10	2000	155.5062	0.000073	0.97786	0.95538
Pb	28	9	2000	124.1062	0.000003	0.99641	0.99788
Cu	29	3	2000	122.3725	0.000030	0.99663	0.99835

**Table 10:** NN-PSO simulation results for the HM models in GW during the WS.

	Hidden Neurons	No. of Particles	No. of Iterations	Elapsed Time (sec)	MSE	R	
						Validation	Testing
Cr	29	2	2000	162.4754	0.00014800	0.96813	0.98011
Cd	27	4	2000	157.0324	0.00003800	0.99426	0.98938
Fe	29	8	2000	145.3954	0.04537300	0.96040	0.97544
Mn	30	8	2000	164.0052	0.00007800	0.98231	0.97926
Zn	28	5	2000	161.1227	0.00012300	0.99861	0.99775
Ni	29	7	2000	160.0693	0.00002200	0.97463	0.98991
Pb	28	10	2000	122.7119	0.00000600	0.98788	0.99495
Cu	30	5	2000	157.5830	0.00000005	0.99925	0.99815

**Table 11:** Optimal parameters of the developed HM models in SW and GW during the DS and WS.

Model	Governing Network Structure	Model	Governing Network Structure
SW Dry Cr	4-30-1	GW Dry Cr	4-30-1
SW Dry Cd	4-28-1	GW Dry Cd	4-28-1
SW Dry Fe	4-29-1	GW Dry Fe	4-29-1
SW Dry Mn	4-27-1	GW Dry Mn	4-30-1
SW Dry Zn	4-30-1	GW Dry Zn	4-29-1
SW Dry Ni	4-29-1	GW Dry Ni	4-27-1
SW Dry Pb	4-30-1	GW Dry Pb	4-28-1
SW Dry Cu	4-30-1	GW Dry Cu	4-29-1
SW Wet Cr	4-29-1	GW Wet Cr	4-29-1
SW Wet Cd	4-30-1	GW Wet Cd	4-27-1
SW Wet Fe	4-28-1	GW Wet Fe	4-29-1
SW Wet Mn	4-29-1	GW Wet Mn	4-30-1
SW Wet Zn	4-30-1	GW Wet Zn	4-28-1
SW Wet Ni	4-27-1	GW Wet Ni	4-29-1
SW Wet Pb	4-29-1	GW Wet Pb	4-28-1
SW Wet Cu	4-28-1	GW Wet Cu	4-30-1

**Figure A1:**
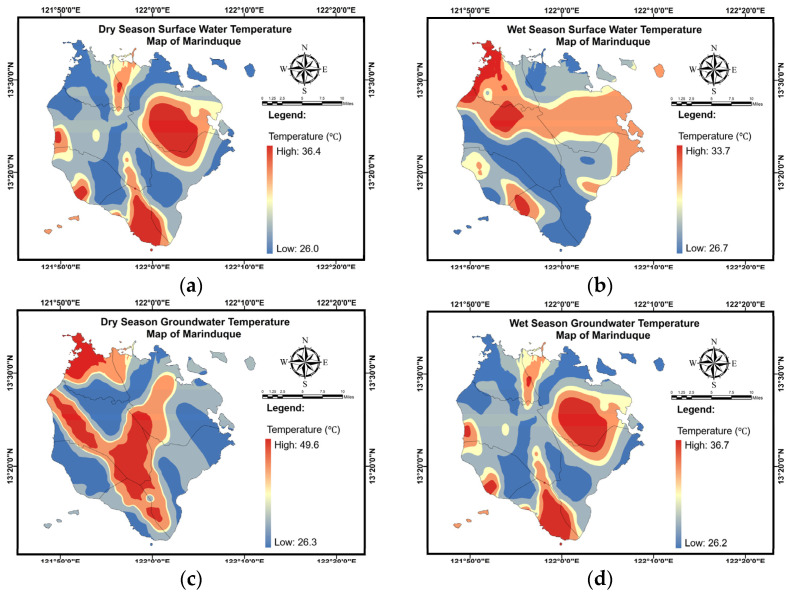
Spatial maps of temperature for (**a**) SW during the DS; (**b**) SW during the WS; (**c**) GW during the DS; (**d**) GW during the WS.

**Figure A2:**
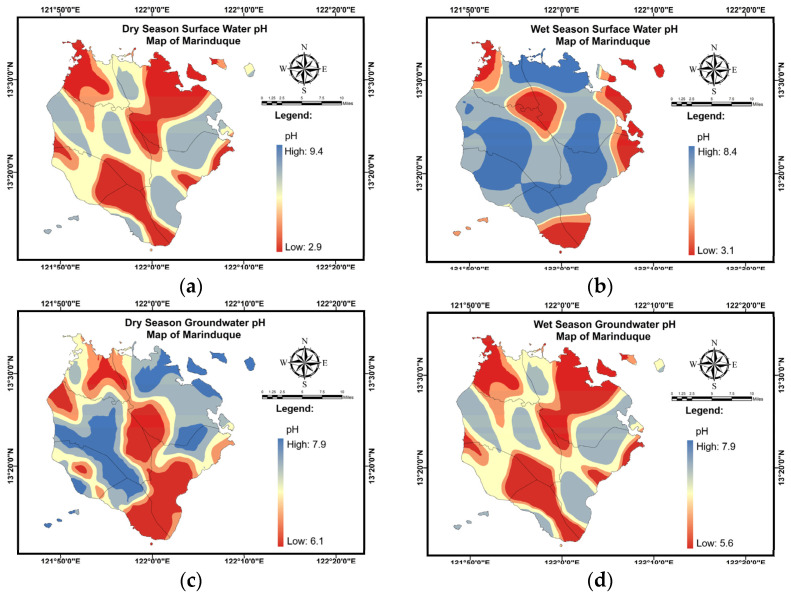
Spatial maps of pH for (**a**) SW during the DS; (**b**) SW during the WS; (**c**) GW during the DS; (**d**) GW during the WS.

**Figure A3:**
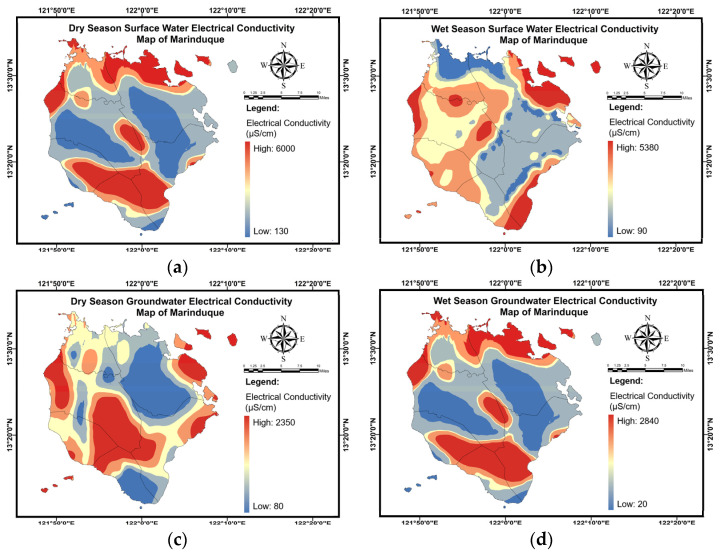
Spatial maps of EC for (**a**) SW during the DS; (**b**) SW during the WS; (**c**) GW during the DS; (**d**) GW during the WS.

**Figure A4:**
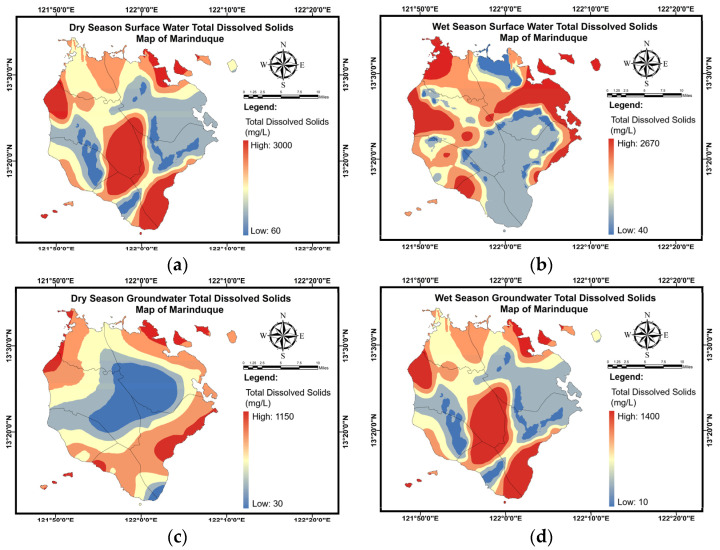
Spatial maps of TDS for (**a**) SW during the DS; (**b**) SW during the WS; (**c**) GW during the DS; (**d**) GW during the WS.

**Figure A5:**
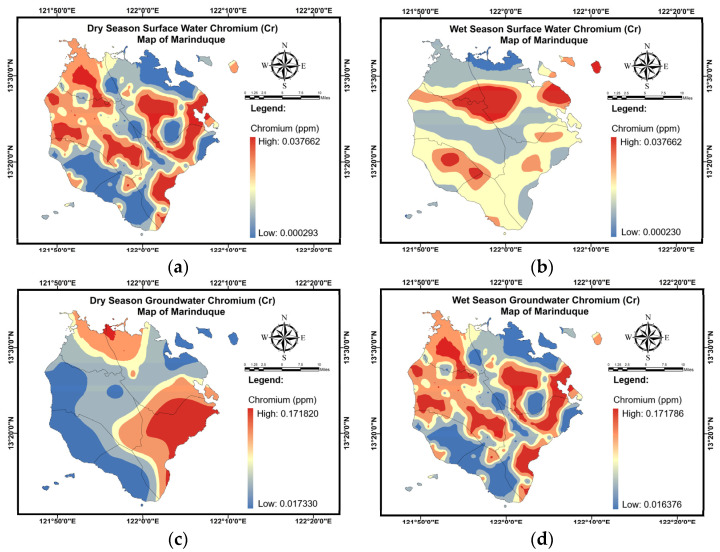
Spatial maps of Cr for (**a**) SW during the DS; (**b**) SW during the WS; (**c**) GW during the DS; (**d**) GW during the WS.

**Figure A6:**
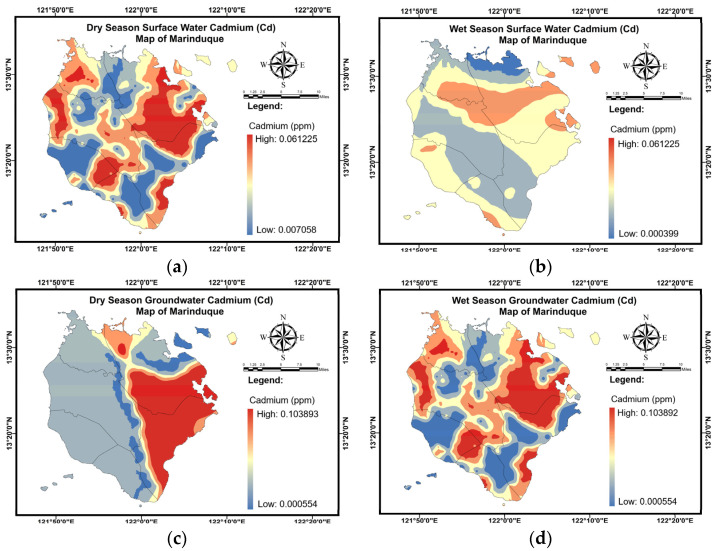
Spatial maps of Cd for (**a**) SW during the DS; (**b**) SW during the WS; (**c**) GW during the DS; (**d**) GW during the WS.

**Figure A7:**
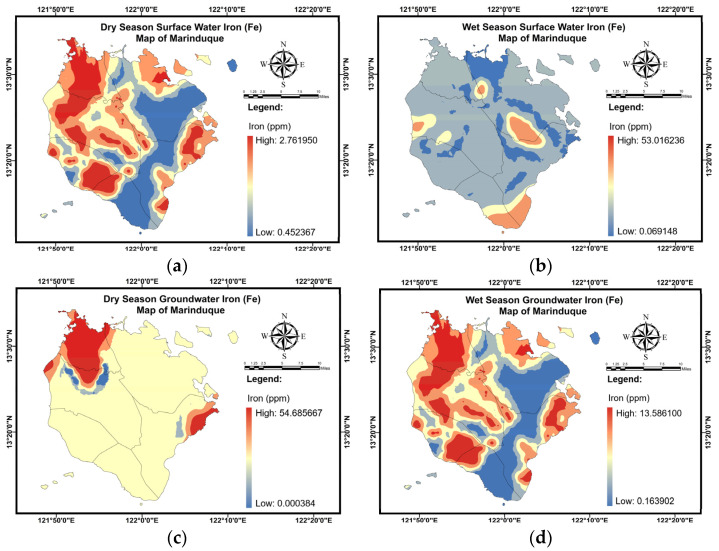
Spatial maps of Fe for (**a**) SW during the DS; (**b**) SW during the WS; (**c**) GW during the DS; (**d**) GW during the WS.

**Figure A8:**
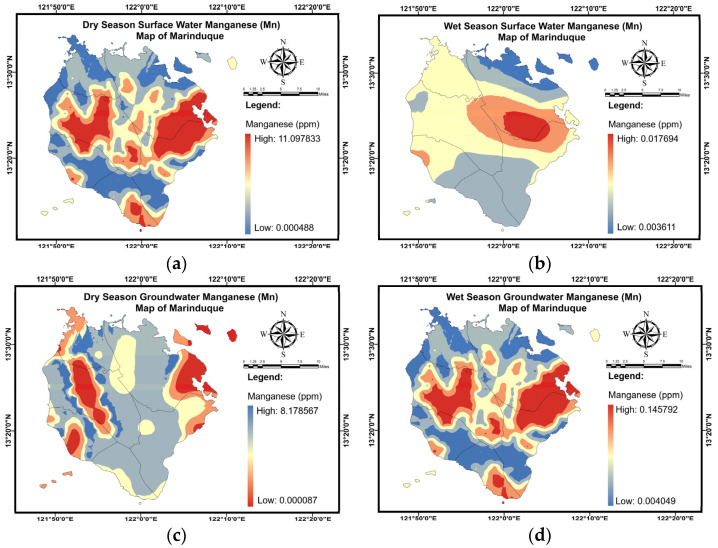
Spatial maps of Mn for (**a**) SW during the DS; (**b**) SW during the WS; (**c**) GW during the DS; (**d**) GW during the WS.

**Figure A9:**
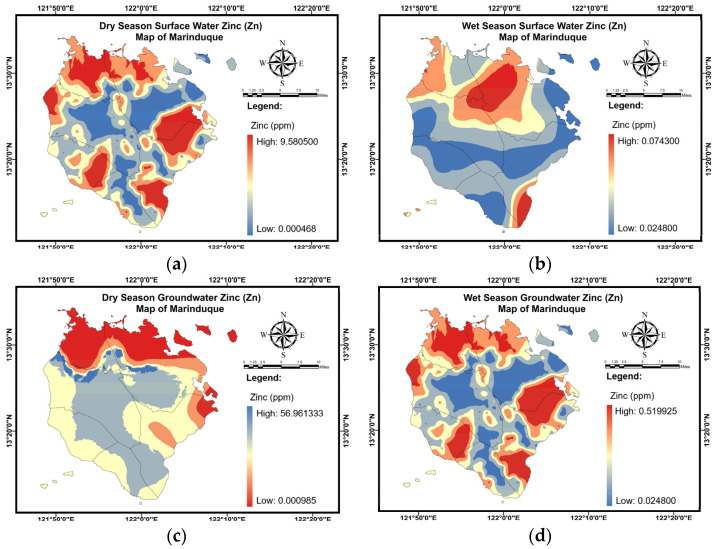
Spatial maps of Zn for (**a**) SW during the DS; (**b**) SW during the WS; (**c**) GW during the DS; (**d**) GW during the WS.

**Figure A10:**
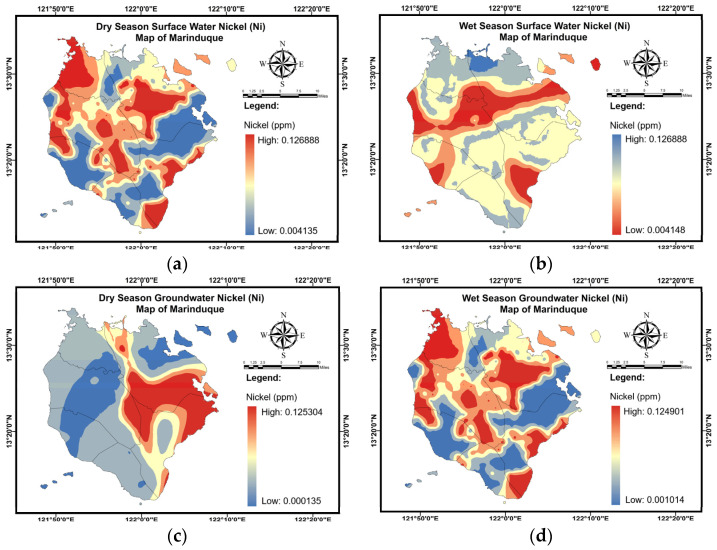
Spatial maps of Ni for (**a**) SW during the DS; (**b**) SW during the WS; (**c**) GW during the DS; (**d**) GW during the WS.

**Figure A11:**
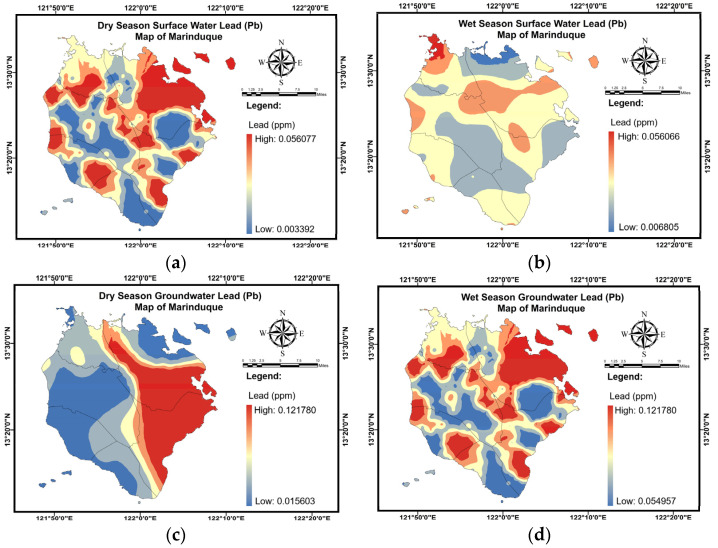
Spatial maps of Pb for (**a**) SW during the DS; (**b**) SW during the WS; (**c**) GW during the DS; (**d**) GW during the WS.

**Figure A12:**
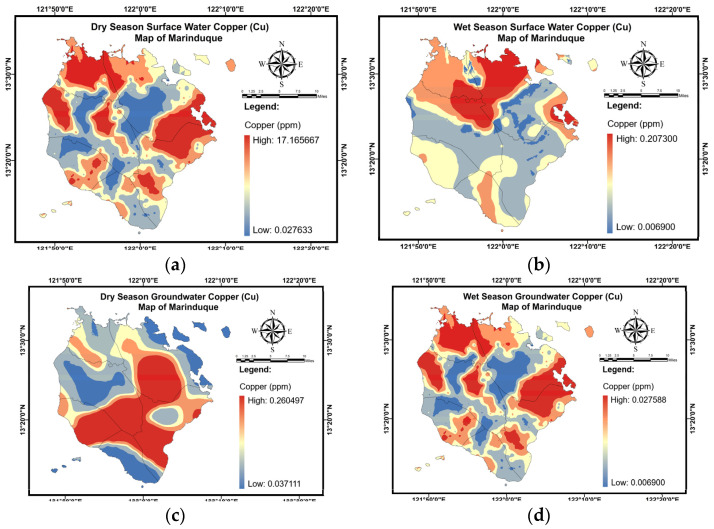
Spatial maps of Cu for (**a**) SW during the DS; (**b**) SW during the WS; (**c**) GW during the DS; (**d**) GW during the WS.

**Figure A13:**
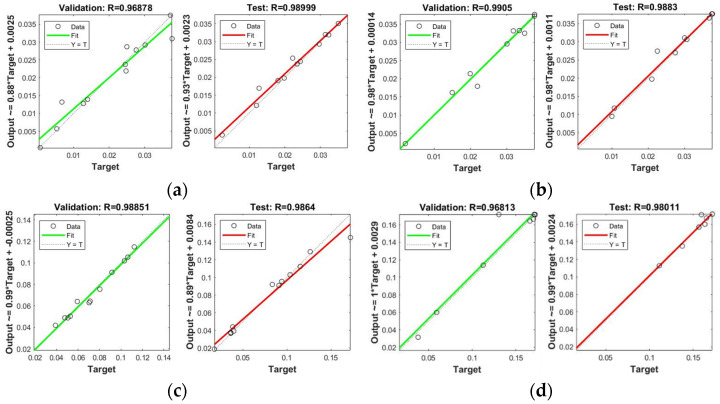
Correlation plots for NN-PSO simulations of Cr for (**a**) SW during the DS; (**b**) SW during the WS; (**c**) GW during the DS; (**d**) GW during the WS.

**Figure A14:**
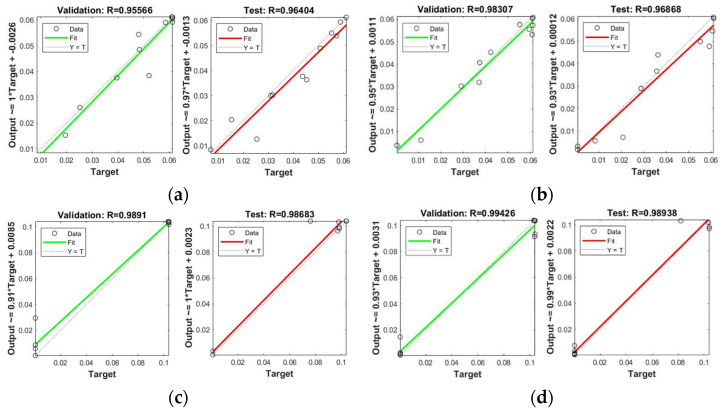
Correlation plots for NN-PSO simulations of Cd for (**a**) SW during the DS; (**b**) SW during the WS; (**c**) GW during the DS; (**d**) GW during the WS.

**Figure A15:**
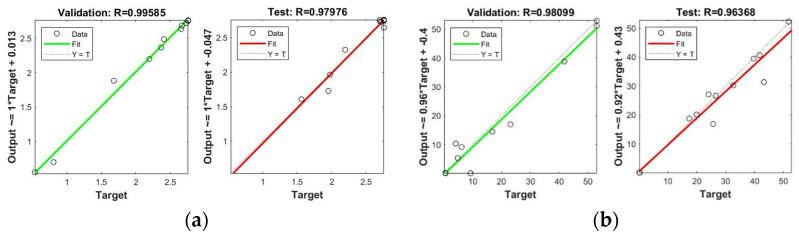

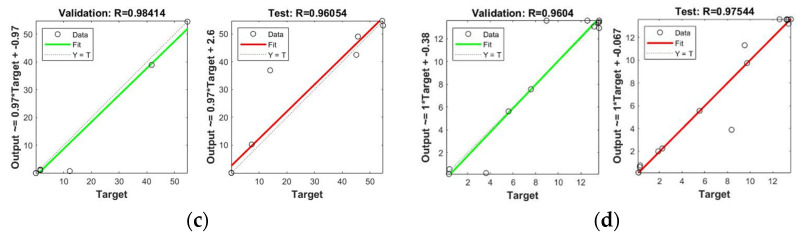
Correlation plots for NN-PSO simulations of Fe for (**a**) SW during the DS; (**b**) SW during the WS; (**c**) GW during the DS; (**d**) GW during the WS.

**Figure A16:**
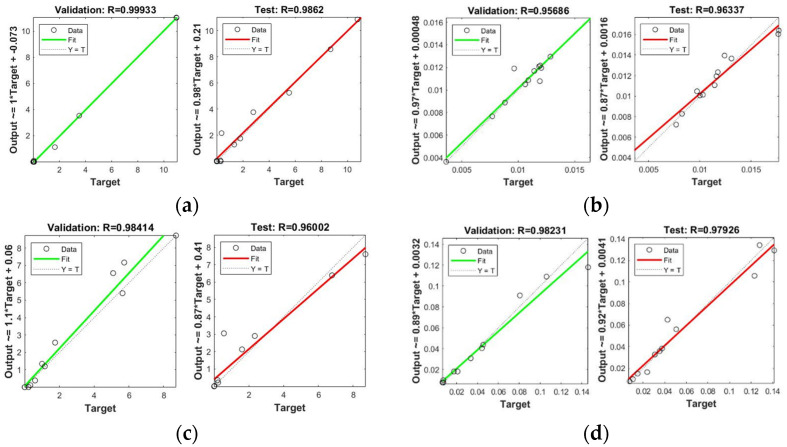
Correlation plots for NN-PSO simulations of Mn for (**a**) SW during the DS; (**b**) SW during the WS; (**c**) GW during the DS; (**d**) GW during the WS.

**Figure A17:**
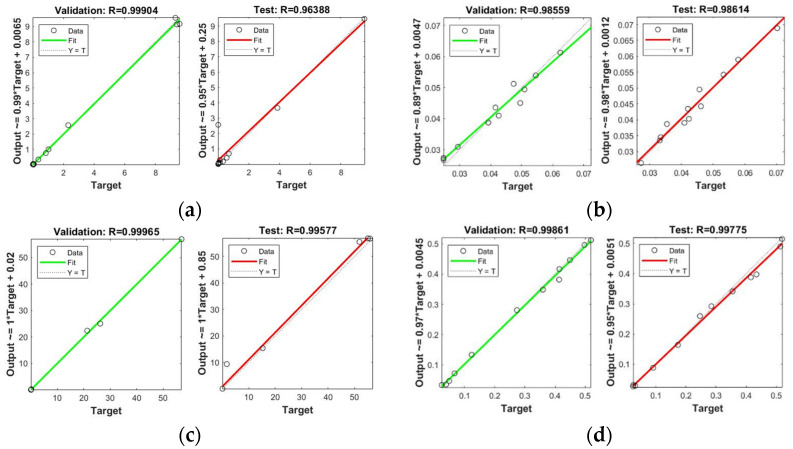
Correlation plots for NN-PSO simulations of Zn for (**a**) SW during the DS; (**b**) SW during the WS; (**c**) GW during the DS; (**d**) GW during the WS.

**Figure A18:**
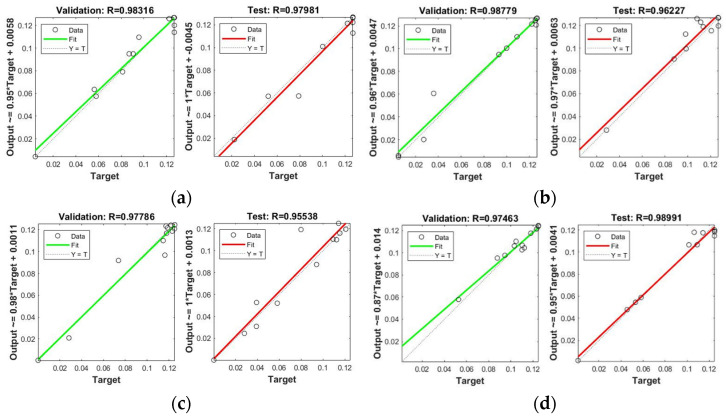
Correlation plots for NN-PSO simulations of Ni for (**a**) SW during the DS; (**b**) SW during the WS; (**c**) GW during the DS; (**d**) GW during the WS.

**Figure A19:**
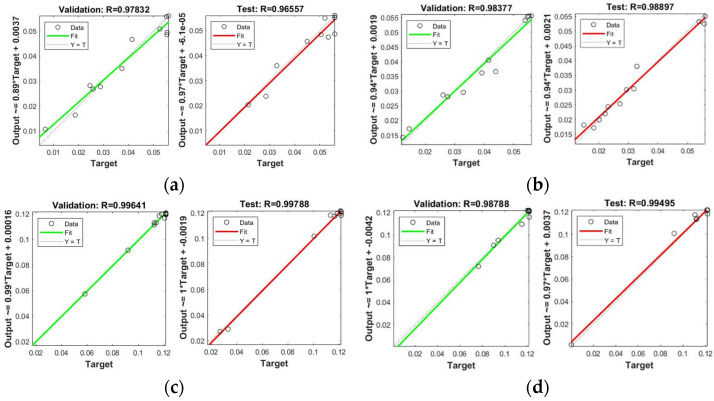
Correlation plots for NN-PSO simulations of Pb for (**a**) SW during the DS; (**b**) SW during the WS; (**c**) GW during the DS; (**d**) GW during the WS.

**Figure A20:**
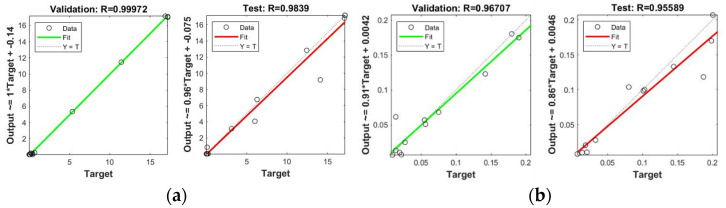

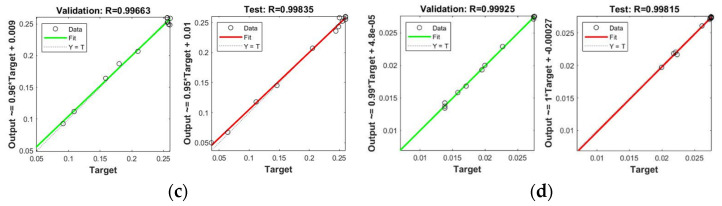
Correlation plots for NN-PSO simulations of Cu for (**a**) SW during the DS; (**b**) SW during the WS; (**c**) GW during the DS; (**d**) GW during the WS.

## Data Availability

All data are contained in the manuscript.

## Appendix A

**Table A1:** Mining disasters in the Philippines.

Location/Date	Cause of Release	Description of Release	Impact	Reference
Siocon, Zamboanga Del Norte/6 April and 11 July 2007	Heavy rains eroded the clay soil and destroyed the concrete wall of the zinc extraction sulphide dam.	Contaminated water with detectable levels of cyanide and mercury ran down the Canatuan River and into the Siocon River, eventually reaching the sea.	Reports of siltation had reached up to 3 m thick, which caused frequent flash floods and obstructed irrigation flow from the river. The river mouth became brown and fish catches decreased.	[118]
Rapu–Rapu Island, Albay/ 11 and 31 October 2005	The tailings pump failed, spilling tailings from the mill’s emergency pond into the gold processing facility and neighboring Alma and Pagcolbon creeks.	At least 20 cubic meters of slurry material (containing cyanide beyond the standard of 0.05 mg/L and other toxic heavy metals and chemicals)	Two kilograms of dead small fish and crustaceans at the shoreline collected on the same day at the location where the affected creeks exit into the sea.	[119]
San Marcelino, Zambales 27 August 27 and 11 September 2002	The spillway of Bayarong tailings dam collapsed during heavy rain.	High concentrations of heavy metals and sulfide materials.	Low-lying settlements were inundated with mining waste, 250 residents were evacuated, and some tailings leaked into Mapanuepe Lake and later into the Sto. Tomas River.	[120]
Sipalay, Negros Occidental 8 December 1995	At the Bulawan gold mine, the pressure of impounded tailings created a leakage in the decant tower of tailings pond no. 1.	Mine tailings caused the siltation of the Sipalay River.	Excessive quantities of dust covered a 5-square-kilometer region, affecting the air quality, and local inhabitants reported a rise in respiratory diseases.	[121]
Toledo City, Cebu 9 August 1999	The outlet of an open pit’s drainage tunnel (from a closed copper mine) was obstructed, resulting in the loosening of accumulated silt and discharge into the Sapangdaku River toward the sea.	5.7 million m^3^ of acidic water	Increased acidity in afflicted water bodies, resulting in fish mortality.	[122]
Placer, Surigao del Norte/ 26 April 1999	Tailings pond No. 7 tailings discharged due to a broken concrete pipe.	700,000 cubic meters of cyanide tailings	Seventeen homes buried, 40 heactares affected, including 20 hectares of agricultural land	[123]
Sibutad, Zamboanga del Norte 6 November 1997	Two strong rain events resulted in mudflows and rockslides into a silt dam.	Sibutad gold project’s silt dam overflowed	Caused flash floods damaging the nearby houses and rice fields and fish kills.	[124]
Mankayan, Benguet 17 October 1986	Tailings pond 3 collapsed as a result of a compromised dam embankment caused by excessive loading.	Mine tailings overflowed and huge amounts of Cu-contaminated mine wastes carried by the Comillas River	Caused siltation of the Abra River, affecting nine towns, and toxic contamination of the river, depriving the region of about 7.33 million kg of rice every year.	[125]
Marinduque Island 6 December 1993	Maguilaguila siltation dam collapsed because of the siltation pressure at the dam wall.	Toxic mine tailings in silt and water	Flooding of the Mogpog River resulted in the death of two children, cattle, contamination of agricultural land, and flooding of downstream communities and Mogpog town.	[126,127]
Marinduque Island 24 March 1996	According to the official explanation, the rock around the plug in the Tapian Pit drainage tunnel was cracked, resulting in the plug’s failure. However, in August 1995, the tunnel began to leak. Marcopper/Placer Dome began drilling 160 m down to the tube in September 1995. The drill struck the tube on 24 March 1996, releasing an air pocket that had been holding back tailings and initiating the leak.	The estimate based on the United Nations is between 2–3 million cubic meters over the first 4–5 days of discharge alone.	Approximately 1200 persons were evacuated, 26 km of the Makulapnit and Boac river systems were rendered impassable by tailings, flash floods cut off five communities, and 67,000 cubic meters of bagged tailings were gathered and placed on the banks of the Boac river since the cleaning started in 2000.	[126,127]

## References

[1] Nem Singh J., Camba A. (2020). The role of domestic policy coalitions in extractive industries’ governance: Disentangling the politics of “responsible mining” in the Philippines. Environ. Policy Gov..

[2] Obasi P.N., Akudinobi B.B. (2020). Potential health risk and levels of heavy metals in water resources of lead–zinc mining communities of Abakaliki, southeast Nigeria. Appl. Water Sci..

[3] Carvalho F.P. (2017). Mining industry and sustainable development: Time for change. Food Energy Secur..

[4] Ali H., Khan E., Ilahi I. (2019). Environmental chemistry and ecotoxicology of hazardous heavy metals: Environmental persistence, toxicity, and bioaccumulation. J. Chem..

[5] Gigantone C.B., Sobremisana M.J., Trinidad L.C., Migo V.P. (2020). Impact of Abandoned Mining Facility Wastes on the Aquatic Ecosystem of the Mogpog River, Marinduque, Philippines. J. Health Pollut..

[6] Long X., Liu F., Zhou X., Pi J., Yin W., Li F., Huang S., Ma F. (2021). Estimation of spatial distribution and health risk by arsenic and heavy metals in shallow groundwater around Dongting Lake plain using GIS mapping. Chemosphere.

[7] Satarug S. (2019). Cadmium sources and toxicity. Toxics.

[8] Wang Y., Su H., Gu Y., Song X., Zhao J. (2017). Carcinogenicity of chromium and chemoprevention: A brief update. OncoTargets Ther..

[9] Boskabady M., Marefati N., Farkhondeh T., Shakeri F., Farshbaf A., Boskabady M.H. (2018). The effect of environmental lead exposure on human health and the contribution of inflammatory mechanisms, a review. Environ. Int..

[10] Alvarez-Bastida C., Martínez-Miranda V., Solache-Ríos M., Linares-Hernández I., Teutli-Sequeira A., Vázquez-Mejía G. (2018). Drinking water characterization and removal of manganese. Removal of manganese from water. J. Environ. Chem. Eng..

[11] Genchi G., Carocci A., Lauria G., Sinicropi M.S., Catalano A. (2020). Nickel: Human health and environmental toxicology. Int. J. Environ. Res. Public Health.

[12] Kim J.J., Kim Y.S., Kumar V. (2019). Heavy metal toxicity: An update of chelating therapeutic strategies. J. Trace Elem. Med. Biol..

[13] Ali M.K., Kim R.Y., Karim R., Mayall J.R., Martin K.L., Shahandeh A., Abbasian F., Starkey M.R., Loustaud-Ratti V., Johnstone D. (2017). Role of iron in the pathogenesis of respiratory disease. Int. J. Biochem. Cell Biol..

[14] Hussain J., Husain I., Arif M., Gupta N. (2017). Studies on heavy metal contamination in Godavari river basin. Appl. Water Sci..

[15] Şahan T., Erol F., Yılmaz Ş. (2018). Mercury (II) adsorption by a novel adsorbent mercapto-modified bentonite using ICP-OES and use of response surface methodology for optimization. Microchem. J..

[16] Diarra I., Kotra K.K., Prasad S. (2021). Application of phytoremediation for heavy metal contaminated sites in the South Pacific: Strategies, current challenges and future prospects. Appl. Spectrosc. Rev..

[17] Ahmed A.N., Othman F.B., Afan H.A., Ibrahim R.K., Fai C.M., Hossain M.S., Ehteram M., Elshafie A. (2019). Machine learning methods for better water quality prediction. J. Hydrol..

[18] Myszczynska M.A., Ojamies P.N., Lacoste A., Neil D., Saffari A., Mead R., Hautbergue G.M., Holbrook J.D., Ferraiuolo L. (2020). Applications of machine learning to diagnosis and treatment of neurodegenerative diseases. Nat. Rev. Neurol..

[19] Hino M., Benami E., Brooks N. (2018). Machine learning for environmental monitoring. Nat. Sustain..

[20] Liu J., Sun Y., Li Q. (2021). High-Resolution PM2.5 Estimation Based on the Distributed Perception Deep Neural Network Model. Sustainability.

[21] Ding X., Zhao Z., Xing Z., Li S., Li X., Liu Y. (2021). Comparison of Models for Spatial Distribution and Prediction of Cadmium in Subtropical Forest Soils, Guangdong, China. Land.

[22] Chen S., Fang G., Huang X., Zhang Y. (2018). Water quality prediction model of a water diversion project based on the improved artificial bee colony–backpropagation neural network. Water.

[23] Jeon J.P., Kim C., Oh B.D., Kim S.J., Kim Y.S. (2018). Prediction of persistent hemodynamic depression after carotid angioplasty and stenting using artificial neural network model. Clin. Neurol. Neurosurg..

[24] Esmaeily H., Tayefi M., Ghayour-Mobarhan M., Amirabadizadeh A. (2018). Comparing three data mining algorithms for identifying the associated risk factors of type 2 diabetes. Iran. Biomed. J..

[25] Chiu C.C., Lee K.T., Lee H.H., Wang J.J., Sun D.P., Huang C.C., Shi H.Y. (2018). Comparison of models for predicting quality of life after surgical resection of hepatocellular carcinoma: A prospective study. J. Gastrointest. Surg..

[26] Bayat H., Ebrahimzadeh G., Mohanty B.P. (2021). Investigating the capability of estimating soil thermal conductivity using topographical attributes for the Southern Great Plains, USA. Soil Tillage Res..

[27] Anifowose F., Ayadiuno C., Rashedan F. (2019). Comparative Analysis of Machine Learning Based Feature Selection Approach for Carbonate Reservoir Cementation Factor Prediction. Proceedings of the International Petroleum Technology Conference.

[28] Torabi-Kaveh M., Sarshari B. (2020). Predicting convergence rate of Namaklan twin tunnels using machine learning methods. Arab. J. Sci. Eng..

[29] Mohandes S.R., Zhang X., Mahdiyar A. (2019). A comprehensive review on the application of artificial neural networks in building energy analysis. Neurocomputing.

[30] Abdallah M., Talib M.A., Feroz S., Nasir Q., Abdalla H., Mahfood B. (2020). Artificial intelligence applications in solid waste management: A systematic research review. Waste Manag..

[31] Sun Y., Zhang J., Li G., Wang Y., Sun J., Jiang C. (2019). Optimized neural network using beetle antennae search for predicting the unconfined compressive strength of jet grouting coalcretes. Int. J. Numer. Anal. Methods Geomech..

[32] Zhang X., Nguyen H., Bui X.N., Le H.A., Nguyen-Thoi T., Moayedi H., Mahesh V. (2020). Evaluating and predicting the stability of roadways in tunnelling and underground space using artificial neural network-based particle swarm optimization. Tunn. Undergr. Space Technol..

[33] Bo L., Yi-Fan Z., Bei-Bei Z., Xian-Qing W. (2018). A risk evaluation model for karst groundwater pollution based on geographic information system and artificial neural network applications. Environ. Earth Sci..

[34] Islam N., Huang W., Zhuang H.L. (2018). Machine learning for phase selection in multi-principal element alloys. Comput. Mater. Sci..

[35] Shariati M., Mafipour M.S., Mehrabi P., Bahadori A., Zandi Y., Salih M.N., Nguyen H., Dou J., Song X., Poi-Ngian S. (2019). Application of a hybrid artificial neural network-particle swarm optimization (ANN-PSO) model in behavior prediction of channel shear connectors embedded in normal and high-strength concrete. Appl. Sci..

[36] Zaman H.R.R., Gharehchopogh F.S. (2021). An improved particle swarm optimization with backtracking search optimization algorithm for solving continuous optimization problems. Eng. Comput..

[37] Alizamir M., Sobhanardakani S. (2018). An Artificial Neural Network-Particle Swarm Optimization (ANN-PSO) approach to predict heavy metals contamination in groundwater resources. Jundishapur J. Health Sci..

[38] Alizamir M., Sobhanardakani S. (2017). Predicting arsenic and heavy metals contamination in groundwater resources of Ghahavand plain based on an artificial neural network optimized by imperialist competitive algorithm. Environ. Health Eng. Manag. J..

[39] Bayatzadeh Fard Z., Ghadimi F., Fattahi H. (2017). Use of artificial intelligence techniques to predict distribution of heavy metals in groundwater of Lakan lead-zinc mine in Iran. J. Min. Environ..

[40] Ghadimi F. (2017). Machine Learning Algorithm for Prediction of Heavy Metal Contamination in the Groundwater in the Arak Urban Area. J. Tethys.

[41] Venkatramanan S., Chung S.Y., Selvam S., Son J.H., Kim Y.J. (2017). Interrelationship between geochemical elements of sediment and groundwater at Samrak Park Delta of Nakdong River Basin in Korea: Multivariate statistical analyses and artificial neural network approaches. Environ. Earth Sci..

[42] Ahangar A.G., Soltani J., Abdolmaleki A.S. (2013). Predicting Mn concentration in water reservoir using Artificial neural network (Chahnimeh1 reservoir, Iran). Int. J. Agric. Crop Sci..

[43] Khudair B.H., Jasim M.M., Alsaqqar A.S. (2018). Artificial neural network model for the prediction of groundwater quality. Civ. Eng. J..

[44] Egbueri J.C., Agbasi J.C. (2022). Data-driven soft computing modeling of groundwater quality parameters in southeast Nigeria: Comparing the performances of different algorithms. Environ. Sci. Pollut. Res..

[45] Singha S., Pasupuleti S., Singha S.S., Kumar S. (2020). Effectiveness of groundwater heavy metal pollution indices studies by deep-learning. J. Contam. Hydrol..

[46] Boudaghpour S., Malekmohammadi S. (2020). Modeling prediction of dispersal of heavy metals in plain using neural network. J. Appl. Water Eng. Res..

[47] Kong G., Wang Q., Huang Q. (2017). Evaluation of groundwater quality in Changping piedmont plain of Beijing based on BP neural network. Trans. Chin. Soc. Agric. Eng..

[48] Said S., Khan S.A. (2021). Remote sensing-based water quality index estimation using data-driven approaches: A case study of the Kali River in Uttar Pradesh, India. Environ. Dev. Sustain..

[49] Ayaz M., Khan N.U. (2019). Forecasting of heavy metal contamination in coastal sea surface waters of the karachi harbour area by neural network approach. Nat. Environ. Pollut. Technol..

[50] Zhang X., Zhang F., Kung H.T., Shi P., Yushanjiang A., Zhu S. (2018). Estimation of the Fe and Cu contents of the surface water in the Ebinur Lake basin based on LIBS and a machine learning algorithm. Int. J. Environ. Res. Public Health.

[51] Lu H., Li H., Liu T., Fan Y., Yuan Y., Xie M., Qian X. (2019). Simulating heavy metal concentrations in an aquatic environment using artificial intelligence models and physicochemical indexes. Sci. Total Environ..

[52] Fattahi H., Agah A., Soleimanpourmoghadam N. (2018). Multi-Output Adaptive Neuro-Fuzzy Inference System for Prediction of Dissolved Metal Levels in Acid Rock Drainage: A Case Study. J. AI Data Min..

[53] Sonmez A.Y., Kale S., Ozdemir R.C., Kadak A.E. (2018). An Adaptive Neuro-Fuzzy Inference System (ANFIS) to Predict of Cadmium (Cd) Concentrations in the Filyos River, Turkey. Turk. J. Fish. Aquat. Sci..

[54] Betrie G.D., Tesfamariam S., Morin K.A., Sadiq R. (2013). Predicting copper concentrations in acid mine drainage: A comparative analysis of five machine learning techniques. Environ. Monit. Assess..

[55] Shakeri Abdolmaleki A., Gholamalizadeh Ahangar A., Soltani J. (2013). Artificial Neural Network (ANN) Approach for Predicting Cu Concentration in Drinking Water of Chahnimeh1 Reservoir in Sistan-Balochistan, Iran. Health Scope.

[56] Aryafar A., Gholami R., Rooki R., Ardejani F.D. (2012). Heavy metal pollution assessment using support vector machine in the Shur River, Sarcheshmeh copper mine, Iran. Environ. Earth Sci..

[57] Gholami R., Kamkar-Rouhani A., Ardejani F.D., Maleki S. (2011). Prediction of toxic metals concentration using artificial intelligence techniques. Appl. Water Sci..

[58] Sharma Y.C., Mukherjee A.K., Srivastava J., Mahato M., Singh T.N. (2008). Prediction of various parameters of a river for assessment of water quality by an intelligent technique. Chem. Prod. Process Model..

[59] Coumans C. (2018). Into the deep: Science, politics and law in conflicts over marine dumping of mine waste. Int. Soc. Sci. J..

[60] Senoro D.B., De Jesus K.L.M., Yanuaria C.A., Bonifacio P.B., Manuel M.T., Wang B.N., Kao C.C., Wu T.N., Ney F.P., Natal P. (2019). Rapid site assessment in a small island of the Philippines contaminated with mine tailings using ground and areal technique: The environmental quality after twenty years. IOP Conf. Ser. Earth Environ. Sci..

[61] Abdel-Satar A.M., Ali M.H., Goher M.E. (2017). Indices of water quality and metal pollution of Nile River, Egypt. Egypt. J. Aquat. Res..

[62] Decker C., Simmons K., United States Environmental Protection Agency (U.S.E.P.A) Operating Procedure for In Situ Water Quality Monitoring (SESDPROC-111-R4). https://www.epa.gov/sites/default/files/2015-06/documents/Insitu-Water-Quality-Mon.pdf.

[63] Migo V.P., Mendoza M.D., Alfafara C.G., Pulhin J.M. (2018). Industrial water use and the associated pollution and disposal problems in the Philippines. Water Policy in the Philippines.

[64] Tolentino P.L.M., Poortinga A., Kanamaru H., Keesstra S., Maroulis J., David C.P.C., Ritsema C.J. (2016). Projected impact of climate change on hydrological regimes in the Philippines. PLoS ONE.

[65] Moodley R., Mahlangeni N.T., Reddy P. (2021). Determination of heavy metals in selected fish species and seawater from the South Durban Industrial Basin, KwaZulu-Natal, South Africa. Environ. Monit. Assess..

[66] Reiman J.H., Xu Y.J., He S., DelDuco E.M. (2018). Metals geochemistry and mass export from the Mississippi-Atchafalaya River system to the Northern Gulf of Mexico. Chemosphere.

[67] De Jesus K.L.M., Senoro D.B., Dela Cruz J.C., Chan E.B. (2021). A Hybrid Neural Network–Particle Swarm Optimization Informed Spatial Interpolation Technique for Groundwater Quality Mapping in a Small Island Province of the Philippines. Toxics.

[68] Chen Y., Yu G., Long Y., Teng J., You X., Liao B.Q., Lin H. (2019). Application of radial basis function artificial neural network to quantify interfacial energies related to membrane fouling in a membrane bioreactor. Bioresour. Technol..

[69] Ostad-Ali-Askari K., Shayannejad M., Ghorbanizadeh-Kharazi H. (2017). Artificial neural network for modeling nitrate pollution of groundwater in marginal area of Zayandeh-rood River, Isfahan, Iran. KSCE J. Civ. Eng..

[70] Okon A.N., Adewole S.E., Uguma E.M. (2021). Artificial neural network model for reservoir petrophysical properties: Porosity, permeability and water saturation prediction. Model. Earth Syst. Environ..

[71] Concha N., Oreta A.W. (2018). A model for time-to-cracking of concrete due to chloride induced corrosion using artificial neural network. IOP Conf. Ser. Earth Environ. Sci..

[72] Mammadli S. (2017). Financial time series prediction using artificial neural network based on Levenberg-Marquardt algorithm. Procedia Comput. Sci..

[73] Rinchon J.P.M. (2017). Strength durability-based design mix of self-compacting concrete with cementitious blend using hybrid neural network-genetic algorithm. IPTEK J. Proc. Ser..

[74] Babu D., Thangarasu V., Ramanathan A. (2020). Artificial neural network approach on forecasting diesel engine characteristics fuelled with waste frying oil biodiesel. Appl. Energy.

[75] El-Gohary K.M., Aziz R.F., Abdel-Khalek H.A. (2017). Engineering approach using ANN to improve and predict construction labor productivity under different influences. J. Const. Eng. Manag..

[76] Abnisa F., Anuar Sharuddin S.D., bin Zanil M.F., Wan Daud W.M.A., Indra Mahlia T.M. (2019). The yield prediction of synthetic fuel production from pyrolysis of plastic waste by levenberg–Marquardt approach in feedforward neural networks model. Polymers.

[77] Alnaqi A.A., Moayedi H., Shahsavar A., Nguyen T.K. (2019). Prediction of energetic performance of a building integrated photovoltaic/thermal system thorough artificial neural network and hybrid particle swarm optimization models. Energy Convers. Manag..

[78] Nguyen H., Bui H.B., Bui X.N. (2021). Rapid determination of gross calorific value of coal using artificial neural network and particle swarm optimization. Nat. Resour. Res..

[79] Tufaner F., Demirci Y. (2020). Prediction of biogas production rate from anaerobic hybrid reactor by artificial neural network and nonlinear regressions models. Clean Technol. Environ. Policy.

[80] Rukhaiyar S., Alam M.N., Samadhiya N.K. (2018). A PSO-ANN hybrid model for predicting factor of safety of slope. Int. J. Geotech. Eng..

[81] da Silva Veloso Y.M., de Almeida M.M., de Alsina O.L.S., Passos M.L., Mujumdar A.S., Leite M.S. (2020). Hybrid phenomenological/ANN-PSO modelling of a deformable material in spouted bed drying process. Powder Technol..

[82] Thio Q.C., Karhade A.V., Ogink P.T., Bramer J.A., Ferrone M.L., Calderón S.L., Raskin K.A., Schwab J.H. (2020). Development and internal validation of machine learning algorithms for preoperative survival prediction of extremity metastatic disease. Clin. Orthop. Relat. Res..

[83] Hannun A.Y., Rajpurkar P., Haghpanahi M., Tison G.H., Bourn C., Turakhia M.P., Ng A.Y. (2019). Cardiologist-level arrhythmia detection and classification in ambulatory electrocardiograms using a deep neural network. Nat. Med..

[84] Disse E., Ledoux S., Bétry C., Caussy C., Maitrepierre C., Coupaye M., Laville M., Simon C. (2018). An artificial neural network to predict resting energy expenditure in obesity. Clin. Nutr..

[85] Gao Z., Zhang H., Mao G., Ren J., Chen Z., Wu C., Gates I.D., Yang W., Ding X., Yao J. (2021). Screening for lead-free inorganic double perovskites with suitable band gaps and high stability using combined machine learning and DFT calculation. Appl. Surf. Sci..

[86] Hou J., Yao D., Wu F., Shen J., Chao X. (2021). Online vehicle velocity prediction using an adaptive radial basis function neural network. IEEE Trans. Veh. Technol..

[87] Zhou F., Liu B., Duan K. (2020). Coupling wavelet transform and artificial neural network for forecasting estuarine salinity. J. Hydrol..

[88] Hesamian G., Akbari M.G. (2020). A robust varying coefficient approach to fuzzy multiple regression model. J. Comput. Appl. Math..

[89] Khademi F., Akbari M., Jamal S.M., Nikoo M. (2017). Multiple linear regression, artificial neural network, and fuzzy logic prediction of 28 days compressive strength of concrete. Front. Struct. Civ. Eng..

[90] Alnowami M., Abolaban F., Hijazi H., Nisbet A. (2022). Regression Analysis of Rectal Cancer and Possible Application of Artificial Intelligence (AI) Utilization in Radiotherapy. Appl. Sci..

[91] Garg M., Dhiman G. (2021). A novel content-based image retrieval approach for classification using GLCM features and texture fused LBP variants. Neural Comput. Appl..

[92] Bhati B.S., Rai C.S. (2020). Analysis of support vector machine-based intrusion detection techniques. Arab. J. Sci. Eng..

[93] Yap K.Y., Sarimuthu C.R., Lim J.M.Y. (2020). Artificial intelligence based MPPT techniques for solar power system: A review. J. Mod. Power Syst. Clean Energy.

[94] Jain U., Nathani K., Ruban N., Raj A.N.J., Zhuang Z., Mahesh V.G. (2018). Cubic SVM classifier based feature extraction and emotion detection from speech signals. Proceedings of the 2018 International Conference on Sensor Networks and Signal Processing (SNSP).

[95] Naicker N., Adeliyi T., Wing J. (2020). Linear support vector machines for prediction of student performance in school-based education. Math. Probl. Eng..

[96] Jimeno-Sáez P., Senent-Aparicio J., Pérez-Sánchez J., Pulido-Velazquez D. (2018). A comparison of SWAT and ANN models for daily runoff simulation in different climatic zones of peninsular Spain. Water.

[97] Tenza-Abril A.J., Villacampa Y., Solak A.M., Baeza-Brotons F. (2018). Prediction and sensitivity analysis of compressive strength in segregated lightweight concrete based on artificial neural network using ultrasonic pulse velocity. Constr. Build. Mater..

[98] Hui E., Stafford R., Matthews I.M., Smith V.A. (2021). Bayesian networks as a novel tool to enhance interpretability and predictive power of ecological models. Ecol. Inform..

[99] Alkadri S., Ledwos N., Mirchi N., Reich A., Yilmaz R., Driscoll M., Del Maestro R.F. (2021). Utilizing a multilayer perceptron artificial neural network to assess a virtual reality surgical procedure. Comput. Biol. Med..

[100] DENR Administrative Order (DAO) No. 2016-08: Water Quality Guidelines and General Effluent Standards of 2016. https://emb.gov.ph/wp-content/uploads/2019/04/DAO-2016-08_WATER-QUALITY-GUIDELINES-AND-GENERAL-EFFLUENT-STANDARDS.pdf.

[101] Tiwari A.K., Singh A.K., Singh A.K., Singh M.P. (2017). Hydrogeochemical analysis and evaluation of surface water quality of Pratapgarh district, Uttar Pradesh, India. Appl. Water Sci..

[102] Huang Z., Zheng S., Liu Y., Zhao X., Qiao X., Liu C., Zheng B., Yin D. (2021). Distribution, toxicity load, and risk assessment of dissolved metal in surface and overlying water at the Xiangjiang River in southern China. Sci. Rep..

[103] Bhuyan M.S., Bakar M.A., Akhtar A., Hossain M.B., Ali M.M., Islam M.S. (2017). Heavy metal contamination in surface water and sediment of the Meghna River, Bangladesh. Environ. Nanotechnol. Monit. Manag..

[104] Wang J., Liu G., Liu H., Lam P.K. (2017). Multivariate statistical evaluation of dissolved trace elements and a water quality assessment in the middle reaches of Huaihe River, Anhui, China. Sci. Total Environ..

[105] Ukah B.U., Egbueri J.C., Unigwe C.O., Ubido O.E. (2019). Extent of heavy metals pollution and health risk assessment of groundwater in a densely populated industrial area, Lagos, Nigeria. Int. J. Energy Water Resour..

[106] Taylor M., Elliott H.A., Navitsky L.O. (2018). Relationship between total dissolved solids and electrical conductivity in Marcellus hydraulic fracturing fluids. Water Sci. Technol..

[107] Ahmed A.S., Sultana S., Habib A., Ullah H., Musa N., Hossain M.B., Rahman M.M., Sarker M.S.I. (2019). Bioaccumulation of heavy metals in some commercially important fishes from a tropical river estuary suggests higher potential health risk in children than adults. PLoS ONE.

[108] Magesh N.S., Chandrasekar N., Elango L. (2017). Trace element concentrations in the groundwater of the Tamiraparani river basin, South India: Insights from human health risk and multivariate statistical techniques. Chemosphere.

[109] Rashid A., Ayub M., Javed A., Khan S., Gao X., Li C., Ullah Z., Sardar T., Muhammad J., Nazneen S. (2021). Potentially harmful metals, and health risk evaluation in groundwater of Mardan, Pakistan: Application of geostatistical approach and geographic information system. Geosci. Front..

[110] Senoro D.B., de Jesus K.L.M., Mendoza L.C., Apostol E.M.D., Escalona K.S., Chan E.B. (2022). Groundwater Quality Monitoring Using In-Situ Measurements and Hybrid Machine Learning with Empirical Bayesian Kriging Interpolation Method. Appl. Sci..

[111] Wagh V.M., Panaskar D.B., Mukate S.V., Gaikwad S.K., Muley A.A., Varade A.M. (2018). Health risk assessment of heavy metal contamination in groundwater of Kadava River Basin, Nashik, India. Model. Earth Syst. Environ..

[112] Bhutiani R., Kulkarni D.B., Khanna D.R., Gautam A. (2016). Water quality, pollution source apportionment and health risk assessment of heavy metals in groundwater of an industrial area in North India. Expo. Health.

[113] Hussein A.A., Chehade A.A. (2020). Robust artificial neural network-based models for accurate surface temperature estimation of batteries. IEEE Trans. Ind. Appl..

[114] Çolak A.B. (2021). A novel comparative investigation of the effect of the number of neurons on the predictive performance of the artificial neural network: An experimental study on the thermal conductivity of ZrO2 nanofluid. Int. J. Energy Res..

[115] Mathew J., Kshirsagar R., Zabeen S., Smyth N., Kanarachos S., Langer K., Fitzpatrick M.E. (2021). Machine Learning-Based Prediction and Optimisation System for Laser Shock Peening. Appl. Sci..

[116] Aydoğdu Ş. (2020). Predicting student final performance using artificial neural networks in online learning environments. Educ. Inf. Technol..

[117] Morin K.A., Hutt N.M. (2001). Prediction of minesite-drainage chemistry through closure using operational monitoring data. J. Geochem. Explor..

[118] Kurita H. (2016). Case Studies of Medium/large-scale Mines in the Philippines (2).

[119] Cotter J., Brigden K. (2006). Acid Mine Drainage: The Case of the Lafayette Mine, Rapu (Philippines).

[120] Lee J.U. (2015). Effect of sulfur concentration on microbial removal of arsenic and heavy metals from mine tailings using mixed culture of *Acidithiobacillus* spp.. J. Geochem. Explor..

[121] Stark J., Li J., Terasawa K. (2006). Environmental Safeguards and Community Benefits in Mining: Recent Lessons from the Philippines.

[122] Ramos H.C., Cabalda M.V., Banaag M.A. Tailings dam accidents and the use of chemicals in mining: Issues, policy response and lessons learned from the Philippines. Proceedings of the International Workshop on Environmental Regulation for Accident Prevention in Mining, Tailings and Chemicals Management.

[123] Holden W.N. (2015). Mining amid typhoons: Large-scale mining and typhoon vulnerability in the Philippines. Extr. Ind. Soc..

[124] Balanay R.M., Halog A. (2017). Promoting life cycle thinking for sustainability in the mining sector of the Philippines. Int. J. Life Cycle Assess..

[125] Cuevas V.C., Orajay J.I., Lagman C.A. (2014). Rice straw compost as amendment to reduce soil copper toxicity in lowland rice paddy field. Philipp. Sci. Lett..

[126] Coumans C. (2000). Canadian Companies in the Philippines: Placer Dome. Undermining the Forests: The Need to Control Transnational Mining Companies: A Canadian Case Study.

[127] Regis E.G. (2006). Assessment of the Effects of Acid Mine Drainage on Mogpog River Ecosystem, Marinduque, Phillippines, and Possible Impacts on Human Communities.

